# Structural and mechanistic diversity in p53-mediated regulation of organismal longevity across taxonomical orders

**DOI:** 10.1371/journal.pcbi.1012382

**Published:** 2025-05-02

**Authors:** Romani Osbourne, Kelly M. Thayer

**Affiliations:** 1 Department of Molecular Biology & Biochemistry, Wesleyan University, Middletown, Connecticut, United States of America; 2 College of Integrative Sciences, Wesleyan University, Middletown, Connecticut, United States of America; Georgia Institute of Technology, UNITED STATES OF AMERICA

## Abstract

The link between p53 tumor suppressive functions and organismal lifespan is multifaceted. Its DNA-repair mechanism is longevity-enhancing while its role in cellular senescence pathways induces pro-aging phenotypes. To understand how p53 may regulate organismal lifespan, cross-species genotype-phenotype (GP) studies of the p53 DNA-binding domain (DBD) have been used to assess the correlation of amino acid changes to lifespan. Amino acid changes in non-DNA-binding regions such as the transactivation (TAD), proline-rich (PRD), regulatory (REG), and tetramerization (TET) are largely unexplored. In addition, existing GP correlation tools such as SigniSite do not account for phylogenetic relationships between aligned sequences in correlating genotypic differences to phenotypes such as lifespan. To identify phylogenetically significant, longevity-correlated residues in full-length p53 alignments, we developed a Python- and R-based workflow, Relative Evolutionary Scoring (RES). While RES-predicted longevity-associated residues (RPLARs) are concentrated primarily in the DBD, the PRD, TET, and REG domains also house RPLARs. While yeast functional assay enrichment reveals that RPLARs may be dispensable for p53-mediated transactivation, PEPPI and Rosetta-based protein-protein interaction prediction suggests a role for RPLARs in p53 stability and interaction interfaces of tumor suppressive protein-protein complexes. With experimental validation of the RPLARs’ roles in p53 stability, transactivation, and involvement in senescence-regulatory pathways, we can gain crucial insights into mechanisms underlying dysregulated tumor suppression and accelerated aging.

## Author summary

Tumor suppressor p53 protein influences lifespan through both DNA repair and cellular senescence pathways. Correlation studies to understand the relationship between differences in p53 orthologs and lifespan have considered only its DNA-binding domain and have not fully incorporated phylogeny in correlation modeling. In this study, we developed and used a computational workflow, Relative Evolutionary Scoring (RES), to identify phylogenetically significant longevity-associated amino acid changes across the full-length p53 protein sequence. Our findings reveal that while most longevity-associated residues are found in the DNA-binding domain, critical residues also exist in other p53 domains. Mutational functional experiments and protein interaction predictions suggest these residues may play a vital role in p53 stability and its interactions with other proteins involved in the induction of senescence. This work broadens our understanding of the mechanisms undergirding dysregulated p53 tumor suppression and its link to accelerated aging.

## Introduction

Usually, aging is a gradual process characterized (in human and mouse models) by molecular biomarkers such as a decrease in leukocyte telomere length, decreased levels of insulin/insulin-like growth factor (IGF-1), and increased inflammation. Several molecular mechanisms have been purported to regulate aging and influence lifespan–many of which have been linked to p53 tumor suppressor activities. In low or high-stress conditions, p53 binds to several target genes and induces tumor-suppressive processes such as DNA repair, apoptosis, and cellular senescence. In a context-dependent manner, its DNA-repair mechanism enhances longevity while aberrant apoptosis and cellular senescence accelerate aging [1].

Genotype-phenotype correlation studies that have sought to map observed differences in lifespan across species to differences in the sequence and structure of p53 ortholog have largely focused on the DNA-binding domain (DBD). Bartas et. al. have linked residual changes in the DBD of several p53 orthologs in kingdom Animalia to longevity. For closely related p53 orthologs, those of longer-lived species possess unique mutations in their DBD that have been hypothesized to enhance their longevity-regulating interactomes. Residues 180–192, which compose the L2 region of the DBD in human p53, are most highly correlated with longevity, leading to the further hypothesis that the unique substitutions in the L2 loops may affect p53’s binding to the minor groove of DNA and induction of cellular processes such as cellular senescence [2].

While the DBD is central to p53’s function as a transcription factor, its transactivation, proline-rich, tetramerization, and regulatory domains are crucial to this function and thus could be important for p53’s regulation of longevity-enhancing and aging-promoting mechanisms. The transactivation domain (TAD) modulates p53’s transactivation of key targets by interacting with proteins that enhance p53’s transcriptional, DNA metabolism, and chromatin modification machinery [3]. p53’s interaction with proteins also modulates its stability, notably the mouse double, minute two (Mdm2) inhibitor protein. Wu et. al. has demonstrated that disruption of the Mdm2-p53 axis accelerates aging in some mice [4]. In another study in which nearly all of their p53 N-terminal was deleted, several mice displayed high resistance to cancer, but also shorter lifespans and accelerated aging phenotypes, suggesting the importance of the Mdm2-p53 axis, and thus the TAD, to the aging process [5]. Relatedly, Mdm2 ubiquitylation of p53 on lysine residues in its regulatory domain (REG) is necessary for its degradation. In addition, the C-terminal which includes both the REG and tetramerization domain (TET) is required for p53’s DNA binding and transactivation mechanisms [6]. This suggests the potential involvement of C-terminal residues on p53’s regulation of longevity. The proline-rich domain (PRD) composing residues 62–92 in the human p53 ortholog modulates p53’s production of and response to caustic elements, such as reactive oxygen species, enabling tight control of p53-mediated apoptosis [7]. The P72 polymorphism in mouse models links the PRD to longevity regulation. The P72 wild type is less active than the mutant R72 and thus predisposes P72 mice to increased cancer risks. Increased activation of the R72 mutant, however, causes increased apoptotic clearance of stem cells, which negatively impacts cellular, tissue, and organismal renewal capabilities. As a result, P72 mice that can escape aberrant tumor development have longer lifespans [8].

Secondly, cross-species genotype-phenotype studies must accurately account for phylogenetic relationships to avoid confounding results. Traits and genetic variations often cluster within closely related species due to shared ancestry rather than functional necessity. Moreover, evolutionary processes are inherently non-independent, so they violate the independence assumption of common correlation models such as ordinary least squares regression. This can lead to biased estimates and inflated Type I errors. By integrating phylogenetically informed methods into genotype-phenotype correlation studies, such as independent contrasts or generalized least squares, we can more accurately account for shared evolutionary histories and produce more robust models [9,10].

Genotype-phenotype studies that seek to correlate p53 residual changes to organismal lifespan must conduct a comprehensive assessment of its full-length sequence and utilize more accurate correlation tools. To address this, we developed a Relative Evolutionary Scoring (RES) workflow to comprehensively investigate the changes in full-length p53 structure across organisms of various taxonomic orders and observed average lifespan. Using the Sorting Intolerant From Tolerant (SIFT) mutation prediction tool and the results from yeast-based functional assays, we characterized the effect of found RES-predicted longevity-associated residues (RPLARs) on p53 function and tumor-suppressive pathways. Finally, we used the PEPPI protein-protein interaction prediction tool to construct a p53-regulated cellular senescence interactome to assess the impact of RPLARs on protein-protein interactions. Altogether, this study elucidates some structural and mechanistic diversity in p53-mediated regulation of longevity. It provides insights into residual clusters linked to longer average lifespans across species, transactivation pathways impacted by mutations at these residues, and a micro protein-protein interactome that may involve interactions at these sites.

## Results

We developed a workflow to quantify the evolutionary changes of single residues of full-length p53 orthologs as a ‘relative evolutionary score’ (RES). The first part of the workflow calculates a position-specific log-odds ratio for each aligned amino acid using a position weight matrix and global background frequencies of amino acids observed [11,12]. This analysis was carried out on seven datasets: first, on a dataset of 386 vertebrate p53 orthologs and then on five datasets of 22–30 orthologs within the taxonomic orders Primates, Rodentia, Artiodactyla, Perciformes, and Carnivora and a seventh dataset comprising the longest organisms from these orders. The sequences were first aligned in the MEGA software package using the MUSCLE alignment algorithm and then scored using RES [13]. Following residue-wise scoring of each sequence in the alignments, we used a phylogenetic generalized least squares model to assess the correlation between RES and average lifespan by taxonomic order, integrating the phylogenetic tree of p53 evolution into the model construction. Residues with p-value < 0.05 were deemed significant and classified as RES-predicted longevity-associated residues (RPLARs).

### RES-average lifespan phylogenetic signal and model fitting

To evaluate the general phylogenetic signal between phylogeny and lifespan, Pagel’s λ was calculated for the 386-vertebrates dataset, the cross-order, and the five order-specific datasets. The 386-vertebrates, Perciformes, Carnivora, and Rodentia datasets had a λ of < 0.0001, suggesting weak phylogenetic signaling. The Primates, Artiodactyla, and cross-order datasets had higher λ of 0.99, 0.75, and 0.55, respectively, suggesting a significantly stronger phylogenetic signaling. These results suggest that phylogeny more strongly explains differences in lifespan among Primates, Artiodactyla, and the cross-order (top five longest-lived species from each order) dataset. To elucidate how well the Brownian motion (corBrownian) model predicts lifespan based on Relative Evolutionary Score (RES), we computed the Akaike information criterion (AIC) and log-likelihood between residual changes of statistically significantly correlated residues and lifespan for all datasets but that of organisms in Carnivora (as they contained no statistically significant residual changes). The Primates model best fitted the observed lifespan and RES, with AIP and log-likelihood of 9.6 and -1.8, respectively. The remaining order-specific and cross-order models all provide better fitting than the 386-vertebrates dataset. The 386-vertebrates dataset model was the least accurate at fitting the observed data, with AIP and log-likelihood of 2680 and -1337, respectively. The correlation model constructed from this dataset finds 112 RPLARs, 13 of which are among the 30 human-aligned RPLARs predicted from the order-specific models. S1 File contains these RPLARs and their associated PGLS fitting statistics. The high AICs and low log-likelihoods suggest that the 386-vertebrates model is not optimal. This poor fit indicates that the 386-vertebrates dataset is too heterogeneous to reliably capture the relationship between RES and lifespan, likely due to the vast evolutionary and ecological differences among vertebrates. Given these limitations, we focus our analysis on taxonomic order-specific datasets, which provide a more controlled evolutionary context and improved model performance.

### RES-average lifespan correlation model is characterized by unique mutations in individual or small groups of longest- or shortest-lived species

**Fig 1:**
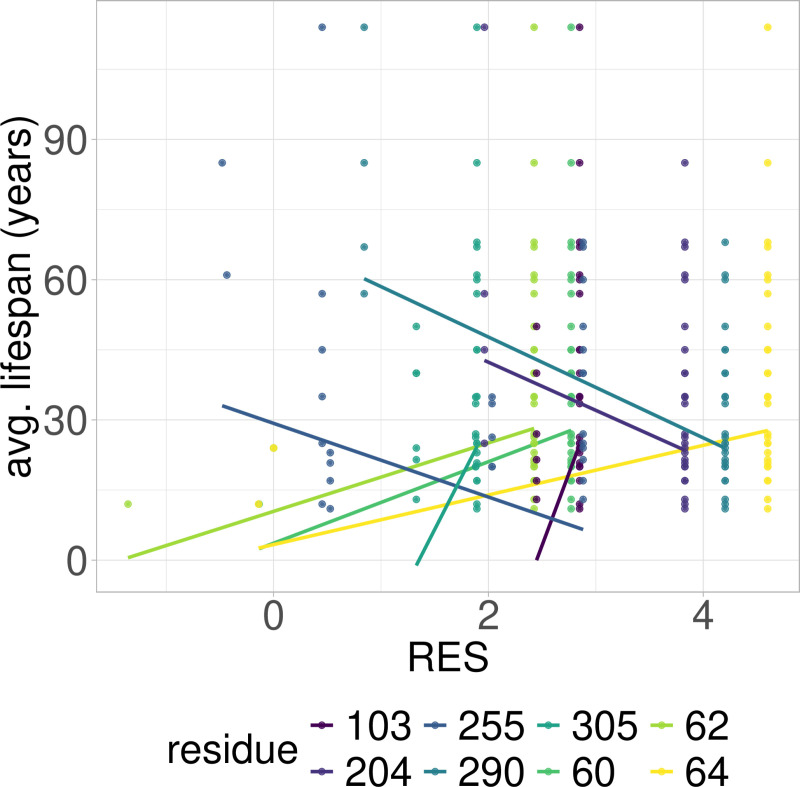
PGLS fitting of average lifespan against RES in Artiodactyla RPLARs. Eight RPLARs found across the TAD and DBD domains.

**Fig 2:**
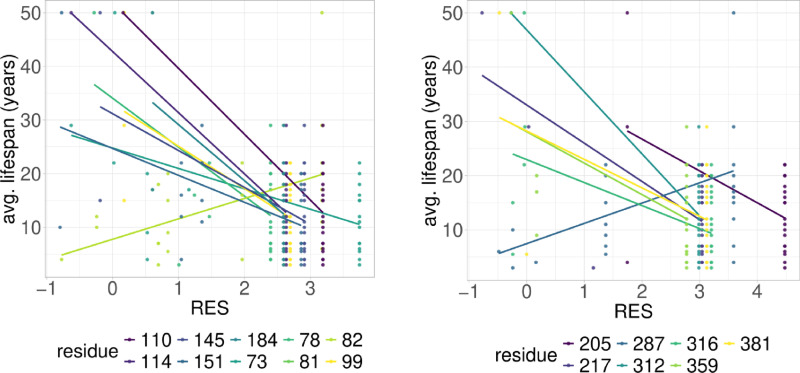
PGLS fitting of average lifespan against RES in Perciformes RPLARs. 17 RPLARs found across TAD, DBD, and REG domains. On left, RPLARs 73-151, and on right RPLARs 205-359.

**Fig 3:**
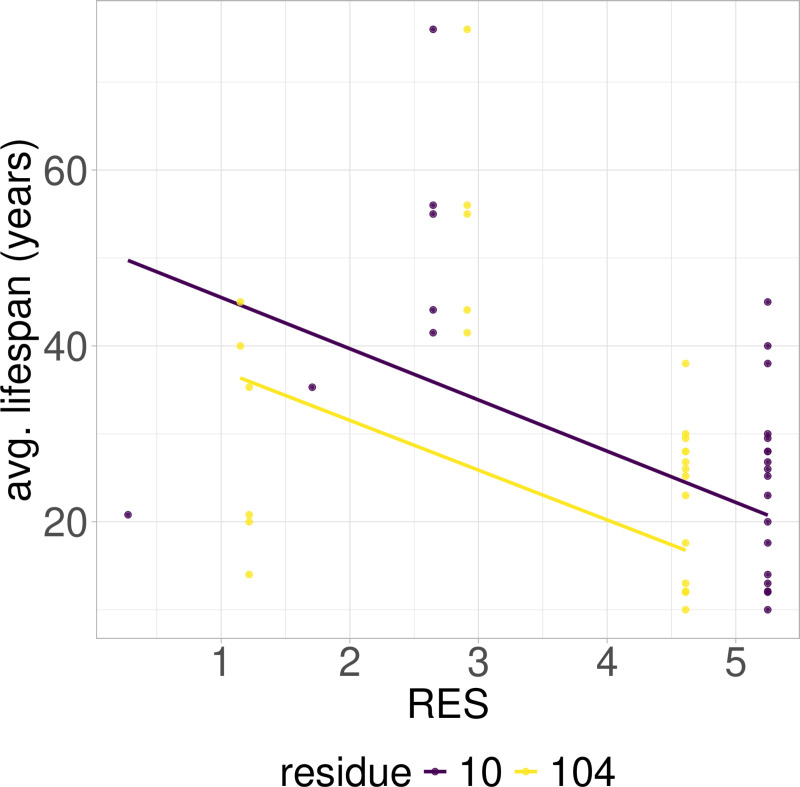
PGLS fitting of average lifespan against RES in Primates RPLARs. Two RPLARs found across the TAD and DBD domains.

**Fig 4:**
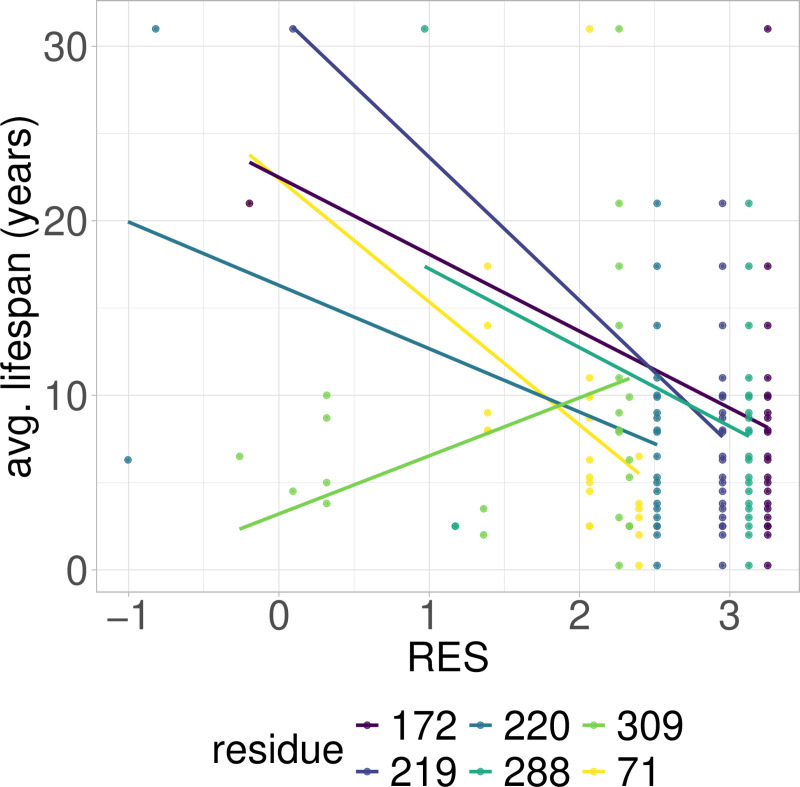
PGLS fitting of average lifespan against RES in Rodentia RPLARs. Six RPLARs found across the TAD and DBD domains.

**Fig 5:**
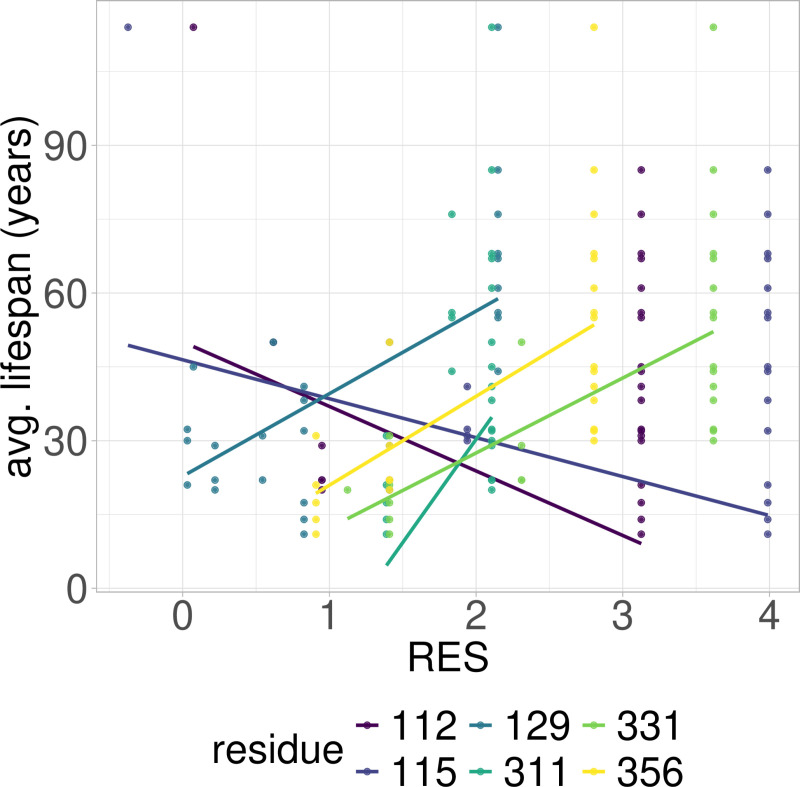
PGLS fitting of average lifespan against RES in cross-order RPLARs. Six RPLARs across the DBD and TET domains.

**Fig 6:**
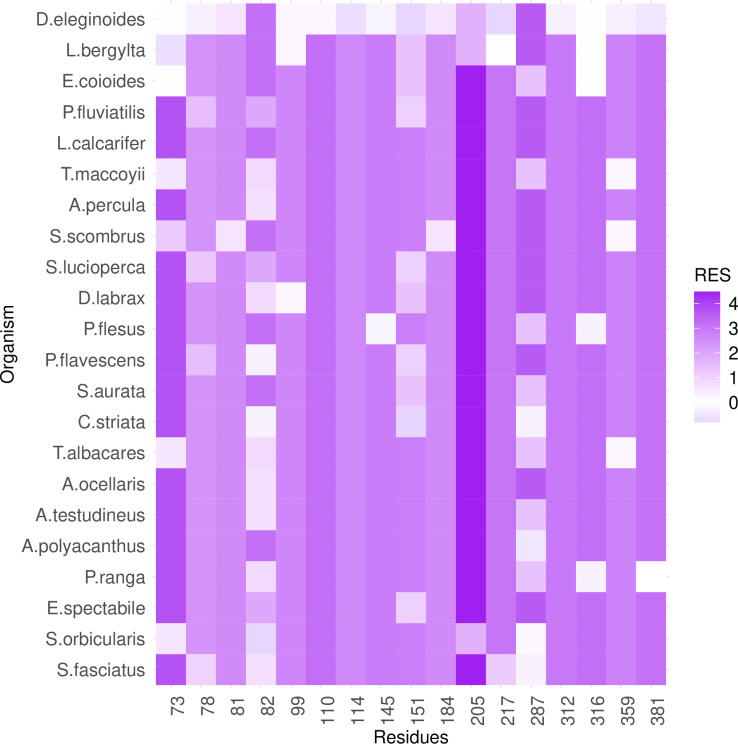
Heatmap of RES changes across Perciformes alignment. Changes in RES at RPLARs characterized by unique substitutions in the longest-lived species D. eleginoides. The y-axis is ordered bottom in descending order of average lifespan.

**Fig 7:**
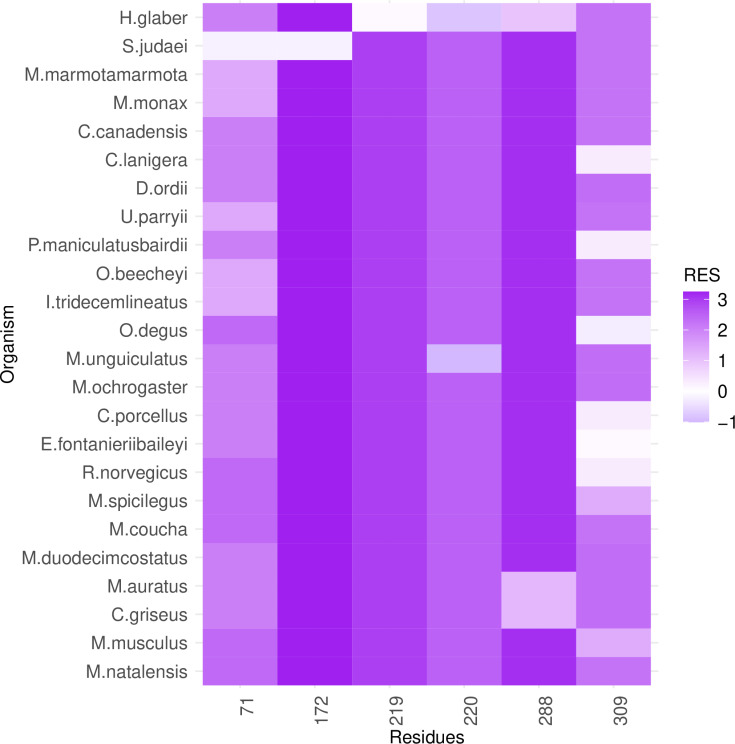
Heatmap of RES changes across Rodentia alignment. Longest-lived H. glaber and S. judaei contain unique substitutions for five out of six RPLARs. The y-axis is ordered bottom in descending order of average lifespan.

**Fig 8:**
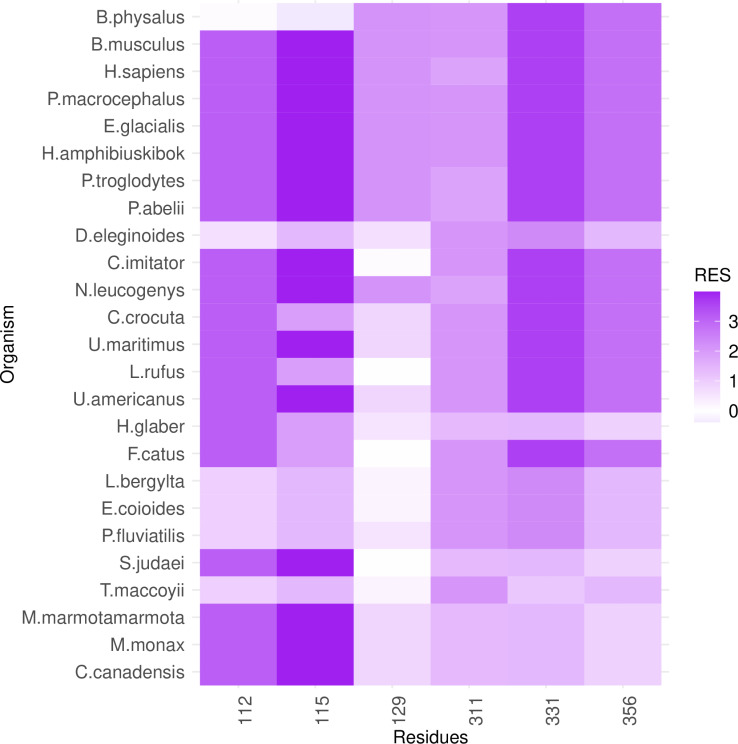
Heatmap of RES changes across cross-order alignment. A mix of unique substitution in longest-lived Balaenoptera physalus and a more equal distribution of RES across organisms of different average lifespans. The y-axis is ordered bottom in descending order of average lifespan.

Out of the 39 RPLARs, 27 have negative correlation coefficients between the RES predictor variable and the average lifespan response variable. The Primates RPLARs have an average correlation coefficient of -5.7, -6.4 for those in Perciformes, -4.1 for those in Rodentia, 12.4 for those in Artiodactyla, and 11.8 for those in the cross-order alignment. Figs 1–5 depicts the phylogenetic generalized least squares fitting of RPLARs within and across orders. Across the models, the group of longest- and shortest-lived organisms have unique substitutions that may explain differences in lifespan. The alignments with negative correlation coefficients differ from those with positive correlation coefficients in that it is their groups of or individual longest species that possess unique substitutions, and thus lower RES. In the latter, it is primarily their shortest-lived species that have unique substitutions. For the Perciformes alignment, only the longest-lived *Dissostichus eleginoides* have unique substitutions at the RPLARs as depicted in Figs 6–8. Correlation coefficients for all RPLARs can be found in the S1 File.

### RPLARs are primarily, not exclusively, found in the DNA-binding domain in order-specific alignments

**Fig 9:**
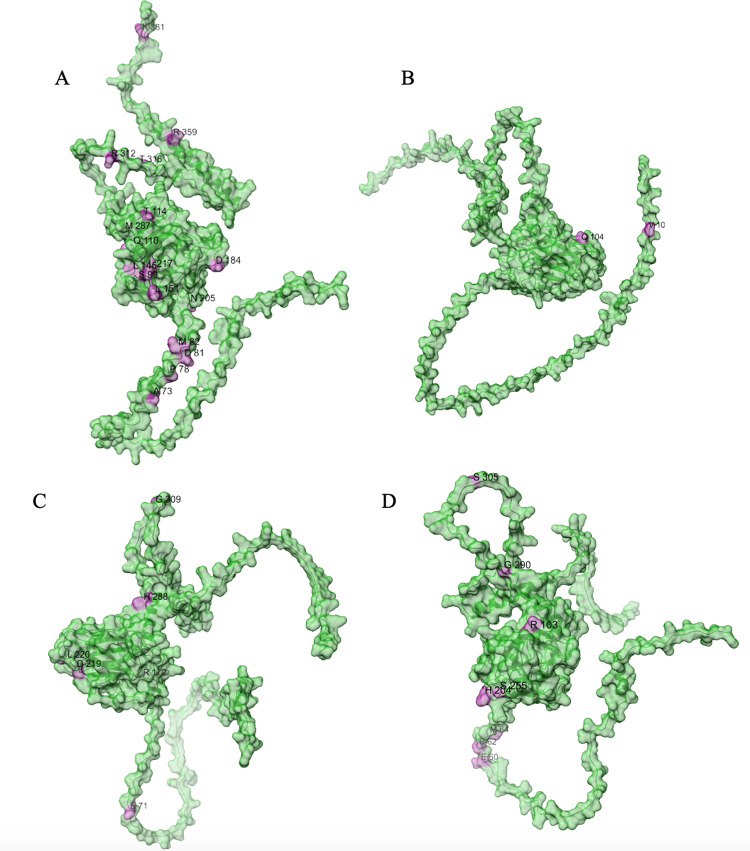
Pymol display of the location of RPLARs in the longest-lived species’ p53 ortholog. AlphaFold2-predicted tertiary structures of (A) *D. eleginoides* ortholog. (B) *H. sapiens ortholog.* (C) *H. glaber* ortholog. (D) *B. phylasus* ortholog. RPLARs are colored pink and labeled with their one-letter amino acid code.

All alignments and correlation modeling reveal that the DNA-binding domain contains the most RPLARs. The RPLARs in each order vary in quantity and location in both the DNA-binding and non-DNA-binding domains of the longest-lived species’ ortholog. The Artiodactyla alignment contains eight RPLARs mapped to the *B. physalus* ortholog: E60 in the TAD2; P62 and M64 in the PRD; R103, H204, S255, and G290 in the DBD, and S305 in the TET domain. The Perciformes alignment contains 17 RPLARs mapped to the *D. eleginoides* ortholog, with nine of these in the DBD: S99, Q110, T114, L145, L151, D184, N205, L217, and M287. Four are found in the PRD: A73, P78, D81, and M82. And the remaining four are equally divided between the two C-terminal domains: R312 and T316 in the TET and R359 and K381 in the REG domain. The Primates alignment contains two RPLARs mapped to the *Homo sapiens* ortholog–residue V10 in TAD1 and Q104 in the DBD. The Rodentia alignment contains six RPLARs mapped to the *H. glaber* ortholog: R172, D219, L220, and H288 in the DBD; E71 in the PRD, and G309 in the TET domain. The Carnivora alignment contains no RPLAR. The cross-order alignment, consisting of the five longest-lived species from each order-specific alignment, contains RPLARs exclusively in the DBD of the *H. sapiens* ortholog: residues G112, H115, A129, N311, Q331, and G356. Fig 9 displays the location of RPLARs in the predicted tertiary structure of the order-specific longest-lived species’ ortholog.

From the alignments, we noted the amino acid changes that affect hydrophobicity or acidity between the shortest-lived and longest-lived species in each taxonomic order. RPLARs in the DBD were primarily found in the L1 and L2 loops as well as in the loops between the hydrophobic β-sandwich core (mapped to *H. sapiens ortholog)* of p53’3 tertiary structure. In the *D. eleginoides* ortholog, hydrophobic residues such as L151 and L217 map to the hydrophilic residues Q128, and H194 of the ortholog of *Salarias fasciatus*, the species with the lowest average lifespan of aligned Perciformes. In the *B. phylasus* ortholog, the acidic residue H204 and the neutral S255 residue map to the basic N199 and R250 residues of the *Ovis aries* ortholog. From the cross-order alignment, and with respect to *H. sapiens* ortholog, L1 residue G112 and S3 residue A129 map to hydrophilic R115 and S135 in the orthologs of the shortest-lived *Thunnus maccoyii* and *Castor canadensis*, respectively. L2 and β-sandwich RPLARs in the Rodentia alignments do not have longest-lived to shortest-lived species mutations that affect hydrophobicity or acidity. RPLARs were found in the *H. sapiens*-like H2 alpha helix, responsible for recognizing and binding the major groove of DNA, across the Perciformes (M287), Rodentia (H288), and Artiodactyla (G290) alignments.

PRD RPLARs were only found in Perciformes, Artiodactyla, and–to a lesser extent–Rodentia alignment. Longest-lived to shortest-lived substitutions at these RPLARs are characterized primarily by hydrophobic to hydrophilic changes and substitution to replace proline residues (A73 to N53, M82 to T62, P78 to M58 in Perciformes and P62 to S61 in Artiodactyl). The PRD of the Artiodactyl alignment is also characterized by deletion events at residues E60 and M64 with respect to the *B. phylasus* ortholog. In these three taxonomic orders, we also found 4 RPLARs in the looped region between the DBD and TET domains, characterized primarily by polar to non-polar changes between longest to shortest-lived orthologs. Only the Perciformes alignment contains RPLARs in its REG domain, housing R to K and K to R substitutions at residues 359 and 381, respectively, the latter being a target of MDM2 ubiquitylation in human and mouse p53 [14]. Four pairs of the RPLARs across the order-specific alignments map to the same residue of the *H. sapiens* ortholog: M82 of D. *eleginoides* and E71 of *H. glaber* map to V73; N205 of *D*. *eleginoides* and H204 of *B. phylasus* map to N210; L215 of *D. eleginoides* and L220 of *H. glaber* map to P222; G309 of *H. glaber* and S305 of *B. phylasus* map to N311–a sight of p53 cleavage and inactivation.

### Missense mutations at RPLARs may have deleterious and non-deleterious effects on p53 function

**Fig 10:**
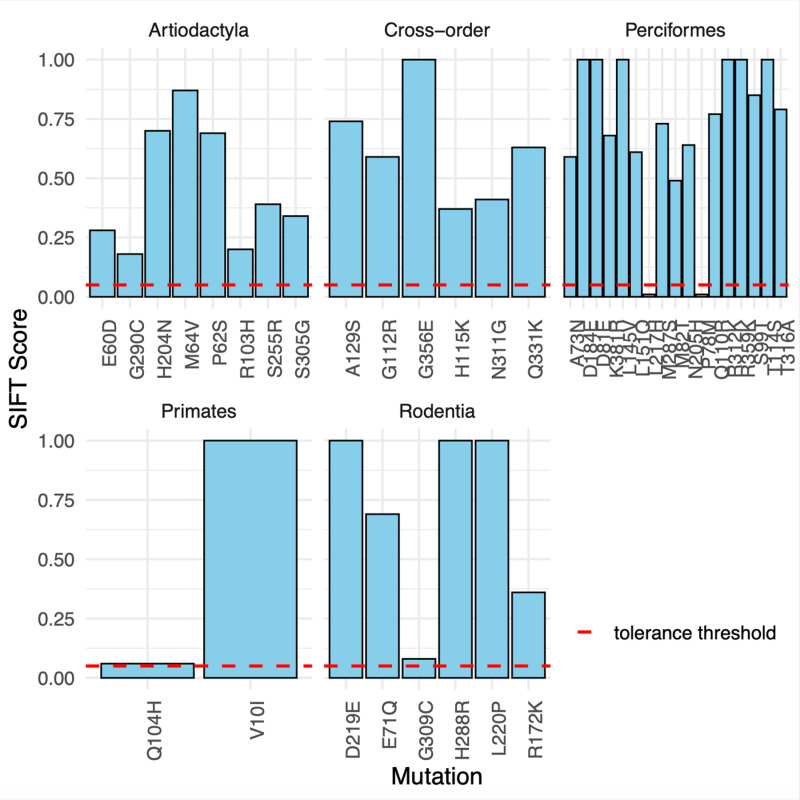
SIFT predictions of mutations at RPLARs in order-specific and cross-order alignments. The Red dashed line represents the SIFT tolerance threshold, which distinguishes deleterious missense mutants from non-deleterious missense mutants.

To assess the effect of mutations at RPLARs on p53 function, we used the Sorting Intolerant from Tolerant (SIFT) algorithm. SIFT uses a sequence homology-based method to predict the likelihood of a residual substitution affecting a protein’s functionality. A SIFT score of 1 suggests that a mutation is not predicted to affect functionality while a score less than 0.05 is predicted to be intolerant; a score between these ranges is predicted to be tolerated. We compared missense mutations of p53 sequences belonging to the longest and shortest-lived organisms for all alignments. If the two sequences had the same residue at a particular point, the organism with the next shortest lifespan and a residual change was tested against the longest-lived organism. The average SIFT score of the longest-lived species’s ortholog due to RPLAR mutation is as follows: *H. sapiens* has an average score of 0.53, *H. glaber* has an average score of 0.67, *B. phylasus* has an average score of 0.46, and *D. eleginoides* has an average score of 0.72. The average SIFT score for RPLARs from the cross-order alignment was 0.62–where mutations were introduced from the shortest-lived *C. canadensis* to the *H. sapiens* ortholog. See Fig 10 for details of individual RPLAR SIFT scoring. Only mutation P78M and L217H, both in the D. *eleginoides* ortholog, were predicted to have a deleterious effect on p53 function. G309C in *H. glaber* p53 and Q104H, a known cancer hotspot, in *H. sapiens* p53 were close to deleterious, with SIFT scores of 0.08 and 0.06, respectively.

### Functional enrichment: Some RPLARs are cancer hotpots, most are dispensable to the transactivation of key targets

**Fig 11:**
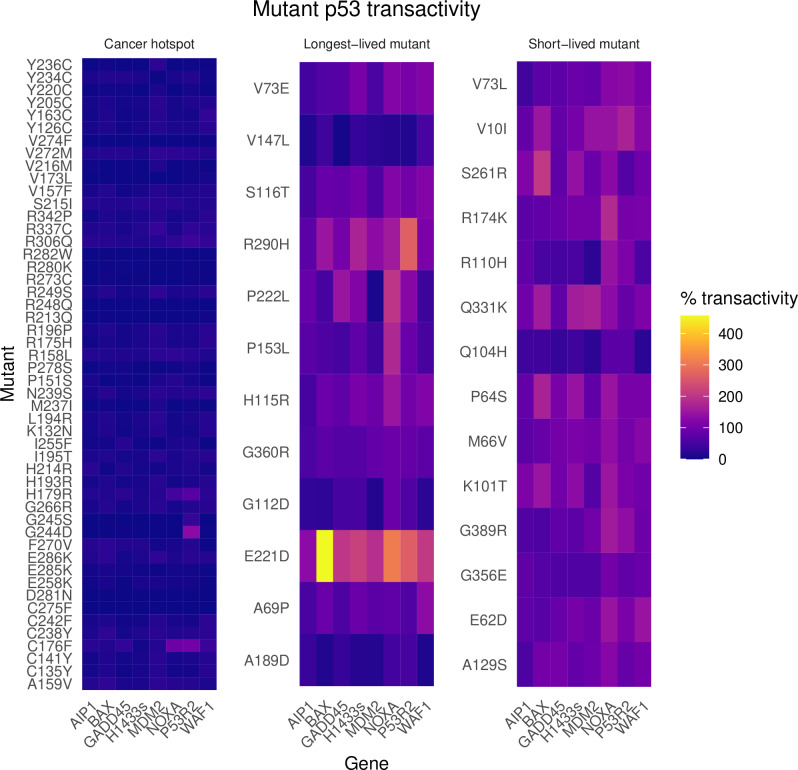
Percentage change of mutp53 transactivation of genes involved in tumor suppression. Heatmap of percent change in mutant p53 transactivation of eight key tumor suppressive genes. Cancer hotpot mutations have a more pronounced effect on transactivation (with respect to wild type) as compared to longest-lived and shortest-lived mutants.

To assess the impact of longevity-correlated mutations on p53’s functions, RPLARs were enriched with Chang et. al’s cancer hotspot dataset, ClinVar, and Kato et. al’s p53 transactivation dataset. Both enrichment analyses suggest that p53-dependent tumorigenesis in humans may be largely independent of RPLARs. *TP53* cancer hotspot enrichment reveals that only five of the 30 human p53-aligned RPLARs are cancer hotspots: Q104H, R110H, S261R, Q317A, and Q331K. Further, pathogenicity enrichment of RPLARs using ClinVar indicates that most human-to-shortest-lived (HS) and human-to-longest-lived (HL) missense substitutions are either benign, likely benign, or of uncertain significance for Li-Fraumeni syndrome. To gain insights into the effects of HS and HL missense mutants on p53 transactivation of key tumor-suppressive genes, the results from Kato et al.’s yeast functional transactivation assay were used to enrich the mutants [15]. To get a baseline control of the change in transactivation that could most significantly compromise p53-mediated tumor suppression, the top 50 p53 cancer hotspot mutations as per Chang et. al’s study were first enriched. On average, these hotspot mutations transactivate the *WAF1, MDM2, BAX, H1433s, AIP1, GADD45, NOXA, and P53R2* promoters only 9.7% of the wild-type (WT) activity, with a range of 0% to 39.7% transactivation. Although *GADD45* has the greatest average decrease in transactivation (5.9% of WT activity), gene-specific transactivation appears to be minor. The HS and HL mutants had an average of 99% and 91% transactivation, respectively. As depicted in Fig 11, of the HS mutants, V10I, Q104H, and Q331K have the greatest average change in transactivation—with V10I and Q331K displaying 125% of WT activity and Q104H displaying 44.3% of WT activity. The fact that both Q104H and Q331K are cancer hotspots suggests that these changes in transactivation may have a direct impact on tumor pathogenesis. For the HS mutants, the greatest change in transactivation was observed in *NOXA* (average 135% of WT). Of the HL mutants, G112D, V147L, A189D, E221D, and R290H have the greatest average change in transactivation—43%, 25.6%, 28%, 248%, and 146% of WT activity, respectively. For the HL mutants, the greatest change in transactivation was observed in *MDM2* and *NOXA* (66% and 130% of WT, respectively). Therefore, when compared to the effect of hotspot mutation on p53 transactivation, HS and HL mutants may be less damaging.

### Cancer hotspot-neighboring RPLARs are key to p53 stability

**Fig 12:**
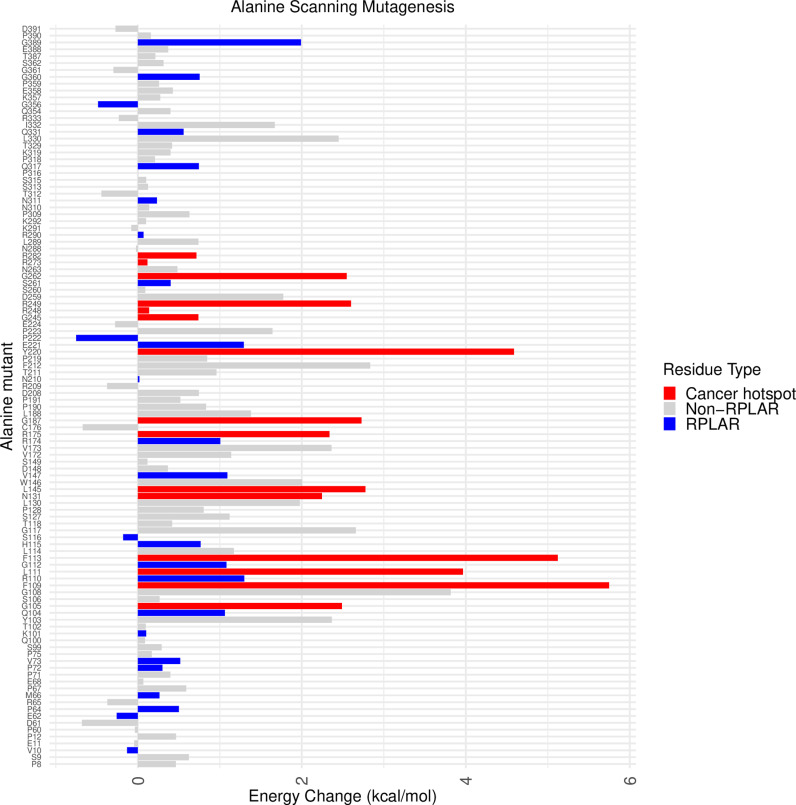
p53 RPLAR alanine scanning ΔΔG. Bar plot of ΔΔG of alanine scanning mutation of cancer hotspots (red), RPLARs (blue), and non-RPLARs (grey).

**Fig 13:**
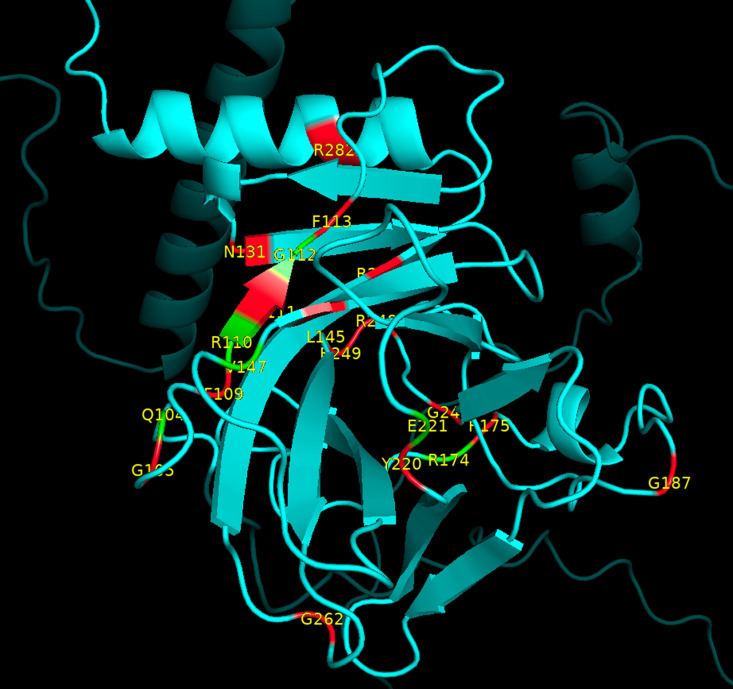
Location of destabilizing RPLARs and cancer hotspots. PyMOL graphics of the p53 DNA binding domain with alanine scanning-induced destabilizing RPLARs (in green), all one residue away from at least one cancer hotspot (in red).

To elucidate how RPLARs may affect the stability of p53, the Cartddg2020 Rosetta protocol was utilized to conduct alanine scanning mutagenesis on AlphaFold3-predicted p53 structure. As a control, the test included 67 non-RPLARs and 15 cancer hotspots in addition to the 30 RPLARs. As Fig 12 shows, cancer hotspot residues have significantly higher ΔΔG (avg. 2.59 kcal/mol) compared to RPLARs and non-RPLARs, avg. 0.61 kcal/mol and 0.49 kcal/mol, respectively. While, on average, alanine mutation at the RPLARs is predicted to be neutral, seven of the thirty RPLARs are predicted to be destabilizing, with an average ΔΔG of 1.26 kcal/mol: residues Q104, R110, G112, V147, R174, E221, and G389. Notably, these residues are adjacent (one amino residue away) from tested cancer hotspots, some such as R110 and G112, “sandwiches” cancer hotspots. Fig 13 maps the relative location of these destabilizing mutants and cancer hotpots on the p53 monomer.

### Some p53-mediated senescence protein-protein interactions may involve RPLARs

**Fig 14:**
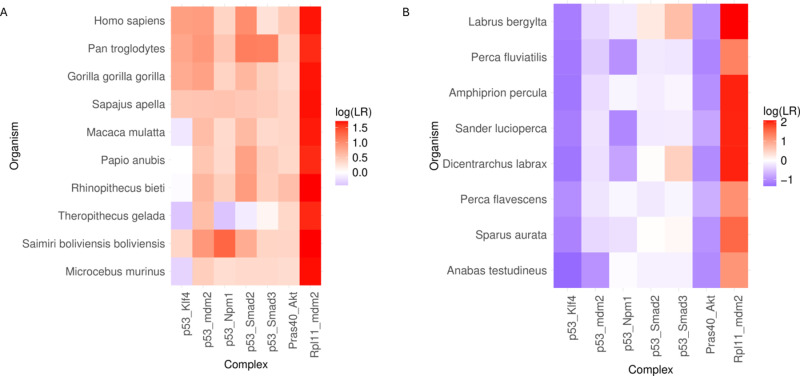
Heatmap of predicted likelihood of p53-mediated cellular senescence interactions. (A) Predicted likelihood of interactions in Primates (organisms ordered from longest-lived to shortest-lived in descending order). p53-Klf4, p53-Smad2, and p53-Smad3 complexes show the greatest difference in log(LR) between species. (B) Predicted likelihood of interactions in Perciformes (heatmap ordered as before). p53-Smad2 and p53-Smad3 complexes show the greatest difference in log(LR) between species.

Based on the increased p53 transactivation of the *p21* promoter following missense mutations at RPLARs, the importance of cellular senescence to the aging process, and the fact that p53 has a complex tumor suppression interactome, we hypothesized that the RPLARs might be involved in p53 interaction with proteins in the p53/p21 cellular senescence pathway [16]. To test this hypothesis, we identified seven protein-protein interactions shown to be involved in this pathway in mouse or human proteins: p53-Klf4, directly involved in the activation of *p21*, p53-Mdm2, p53-Smad2, p53-Smad3, p53-Npm1, Akt-Pras40, and Rpl11-Mdm2 [17–24]. Since the Perciformes alignment produced the highest number of RPLARs, we used PEPPI to construct and compare quantitatively the likelihood of interactions in a group of Perciformes and Primates of varying lifespans. Fig 14 shows the difference in these interactions between the two orders. Most of the interactions are not predicted to be shared between organisms of the two orders–the only fully shared interaction is between Rpl11 and Mdm2, a cellular senescence-inducing interaction. Among the Primates, the orthologs of the longer-lived *H. sapiens* and *Pan troglodytes* have a higher likelihood of interaction for the p53- Klf4, Mdm2, Smad2, and Smad3 interactions while the orthologs of the shorter-lived *Saimiri boliviensis* have the highest likelihood of interaction for the p53-Nmp1 interaction. A similar trend is observed for the longest-lived Perciforme, *Labrus bergylta*, having the highest likelihood of interaction for the p53-Smad2 and p53-Smad3 interactions. S3 File in the Supporting Information section lists the log(LR) values for each of these interactions across all species included in the study.

To test whether the RPLARs may be involved in the p53- Smad2, Smad3, Klf4, and Npm1 interactions, we used AlphaFold to predict each dimeric complex between the orthologs shown to have the likelihood of interaction in each order: we predicted p53-Smad2 and p53-Smad3 complexes for *P. troglodytes* and *L. bergylta* orthologs, the p53-Npm1 complex for *S. boliviensis* ortholog and the p53-Klf4 complex for *H. sapiens* ortholog. We then used the interfaceResidue PyMol module to predict potential interacting residues in the predicted complexes. While interface residues are found in the N- and C- terminals, the majority are found in L2, L3, and to a lesser extent L1, loops of the DBD as depicted in S1 Fig. Three p53 interface residues aligned to the RPLARs of the *H. sapiens* ortholog: K71 and Q294 of the p53-Npm1 complex aligned to K101 and Q294, respectively and V10 of the p53-Klf4 complex aligned to V10.

## Discussion

Existing genotype-phenotype correlation models of p53 sequences to lifespan do not fully account for its non-DNA binding domains nor the non-independence of evolution in model fitting. Here, we have presented a genotype-phenotype correlation workflow, RES, that correlates full-length p53 residual evolutionary changes to the average lifespan of 126 p53 orthologs across five taxonomic orders. To accurately model lifespan based on quantified residual changes, we used phylogenetic generalized least squares which take into account the evolutionary relationship of p53 orthologs. To elucidate some of the biochemical and molecular significance of the RES-predicted longevity-associated residues (RPLARs), we analyzed the results of yeast functional assays from the NCI and used SIFT, Rosetta’s Cartddg20202 protocol, and PEPPI to predict the RPLARs’ effect on p53’s function, stability, and involvement in protein-protein interactions in the p21/p53 cellular senescence pathway.

The seven sets of organisms being used to model the relationship between RES and average lifespan have varying phylogenetic signals and model fit accuracy. The all-Vertebrates, Carnivora, Rodentia, and Perciformes datasets show little to no phylogenetic signal (λ < 0.0001), suggesting that phylogeny may play a minimal role in shaping p53 evolution among these taxa. This implies that variation in p53 within these groups is likely driven more by selection pressures unrelated to shared ancestry, such as environmental or functional constraints. The Primates, Artiodactyla, and cross-order alignments exhibit significantly stronger phylogenetic signals (λ > 0.5), indicating that p53 variation in these groups is more closely linked to evolutionary history. This suggests that shared ancestry contributes meaningfully to p53 evolution, and differences in lifespan and p53 function within these clades may be partially explained by phylogenetic relationships. In addition to the low phylogenetic signal, the all-Vertebrates dataset does not accurately model RES-average lifespan relation under the Brownian motion model. The taxonomic order-specific groups more accurately model this relation with more favorable AICs and log-likelihoods. Thus, our analyses of the relationship between p53 sequence variations and lifespan prioritize the taxonomic order-specific models.

RPLARs vary by taxonomic order. The two in the Primates alignments are in the TAD1 and DBD; the Perciformes alignment, although it contains most RPLARs in its DBD, also houses four in its PRD and two REG domains; the Rodentia alignment contains one in its PRD and the others in its DBD; the Artiodactyla alignment contains three in its PRD and three in its DBD. The Carnivora alignment contains no RPLARs, and all but the Primates alignment also contain RPLARs in the spacer, looped region between the DBD and TET domains. This diversity in both domain and number of RPLARs per order suggests that p53-mediated regulation of longevity may be order- and context-specific, a characteristic feature of the transcription factor [25]. Still, RLARs being found in the DBD and PRD across multiple alignments may point to shared mechanisms of longevity regulation. DBD RPLARs mapped to the human ortholog reside primarily in the L1, L2, and intra β-sheet loops. In these lopped regions, RPLARs may play an important role in DNA recognition, binding, and maintaining p53’s structural integrity [26]. RPLARs within the PRD and primarily around P72–a site of a lifespan-enhancing polymorphism in mouse models–could play crucial roles in apoptotic regulation, as disruption of the PxxP motifs could inhibit p53’s binding to the SH3 family, apoptosis-regulating proteins [7,27].

The shared mapping of residues V73, N210, P222, and N310 of the human p53 ortholog to multiple non–-Primates alignment may underscore similarities in longevity regulation across orders. As V73 is among the clusters of N-terminal residues whose conformational dynamics are most affected by the presence of SNPs, it may play an important role in modulating p53’s interaction with coactivators and inhibitors [28]. Its proximity to the P72, whose R72 polymorphism stabilizes p53, suggests that it may be implicated in regulating p53’s stability. ^1^H-^15^N spectra analysis also reveals a significant chemical shift on residue N210, suggesting that it may be a DNA-contacting residue or be close to such residues [29]. DBD mutations detected in glioblastomas and hepatocellular carcinoma cause p53 to undergo liquid-liquid separation. Interestingly, RPLAR P222 accelerates LLSP in the M237I mutant by increasing hydrophobic interactions near the L3 loop [30]. The fourth of the commonly mapped residues, N311, is the site of asparaginyl endopeptidase cleavage, which leads to p53 degradation [31]. While these biochemical functions and biophysical properties do not translate to direct residue-specific p53-mediation of a longer lifespan, they give insights into potential mechanisms undergirding differential regulation of p53’s tumor suppressive activities and potential pathways for dysregulation. Murine models harboring mutants at these residues could be used to assess the physiological effects of these differences.

*H. sapiens*, *B. phylasus, D. eleginoides, and H. glaber* have the highest average lifespans, while *C. jacchus*, *O. aries*, *S. fasciatus*, and *M. natalensis* have the lowest average lifespans in their respective taxonomic orders. Our workflow reveals that these species have unique RPLAR substitutions that are significantly correlatable to increased (for longer-lived species) or decreased (for shorter-lived species) lifespans. Lio et. al. have shown that the p53-mediated apoptotic genes such as *CASP3*, *CASP8*, and *TP73* are either positively selected or duplicated in the longest-lived Artiodactyl species, implicating apoptosis in longevity regulation [32]. *H. glaber* possesses a hyperstable p53 which offers the organism enhanced cancer resistance. Our analysis of p53 mutation functional data provides further insights into the potential roles of RPLARs in regulating the stability and transactivation of a pro-apoptotic and senescence gene *p21*.

The RPLAR mutants Q104H, R110H, S261R, Q317A, and Q331K are cancer hotspots, but their effects on p53 transactivation are less pronounced than the more common cancer hotspots. Transactivation enrichment analysis reveals that RPLARs may be dispensable for direct p53 transactivation of *MDM2*, *NOXA*, *WAF1*, *AIP1, WAF1, H1433s, and GADD45*. Non-RPLAR cancer hotspot p53 mutants significantly aberrate transactivation of these targets, reducing transactivation by an average of over 90% compared to WT p53. Human to longest-lived (HL) and human shortest-lived (HS) p53 mutants reduce transactivation by an average of less than 10% of WT. Still, some RPLAR cancer hotpots and non-hotspots have comparable effects on transactivation. Mutant Q104H, a cancer hotspot RPLAR mutant, reduces the transactivation of the eight targets by an average of 60% compared to WT P53. RPLAR HL mutants G112D, V147L, and A189D, although not cancer hotspots, also aberrate p53 transactivation of the above targets comparably to cancer hotspots. Interestingly, the HL mutant E221D increases transactivation by nearly 2.5 times that of WT p53. The results of Rosetta-based Cartddg2020 alanine scanning give insights into how some of these RPLARs may be impacting p53 structure. The results suggest that mutations at the cancer hotpot RPLARs Q104 and R110 may unfavorably impact p53 stability. Mutations at five other RPLARs, including V147 and E221 may also favorably impact p53 stability. Notably, all of these residues are within 1 residue away from known cancer hotspots. These findings suggest that while RPLAR cancer hotspot mutants generally have a less pronounced effect on p53 transactivation compared to other cancer-associated p53 mutations, specific RPLAR mutations can still significantly impair or enhance transactivation. The observed proximity of destabilizing and stabilizing RPLARs to known cancer hotspots highlights a potential structural basis for their functional effects, reinforcing the idea that local destabilization near key regulatory sites may modulate p53 activity. Additionally, the distinct behavior of HL mutants, particularly E221D’s enhancement of transactivation, suggests that some longevity-associated p53 variants may retain or even enhance tumor-suppressive functions, potentially contributing to extended lifespan phenotypes. As per the results of our PEPPI study, p53-mediated cellular senescence regulation may vary across species of Primates and Perciformes. The shared p53-Smad2 and p53-Smad3 interactions, while not predicted to directly involve RPLARs, implicate the Smad family of proteins in the p53-mediated regulation of longevity across orders. Their roles as substrates in the TGF-β pathway, which cross-talks with p53 mechanisms of tumor suppression, point to a complex regulatory mechanism [33–37].

## Conclusions & future directions

This study introduces RES, a computational framework that correlates full-length p53 residual evolutionary changes with organismal lifespan using phylogenetic generalized least squares. Our results highlight the importance of taxonomic order-specific modeling in capturing the relationship between p53 sequence variation and lifespan, as broad Vertebrate-wide models show weak phylogenetic signals and poor fit. The identified RPLARs exhibit taxon-specific distributions, yet commonly cluster in the DBD and PRD, regions critical for DNA binding, structural integrity, and apoptotic regulation. Functional analyses using yeast assays, SIFT, Rosetta’s Cartddg2020, and PEPPI reveal that RPLARs modulate p53 stability, transactivation, and protein-protein interactions, particularly in the p21/p53 senescence pathway. The observed proximity of RPLARs to known cancer hotspots suggests potential structural mechanisms underlying their role in longevity regulation.

Computational and experimental techniques could be used to validate and further clarify the roles of RPLARs in p53-mediated regulation of longevity on molecular and physiological levels. Molecular dynamics simulations could probe potential allosteric signaling mechanisms mediated by RPLARs to provide insight into long-range conformational changes and intramolecular communication within p53. Co-immunoprecipitation assays could identify interactions between RPLAR-modified p53 and co-regulatory proteins, such as Smad2/3, Klf4, and Npm1, to clarify their role in apoptosis and senescence pathways. *In vivo* murine models engineered with human-like RPLAR substitutions may enable investigations into how RPLARs influence cancer resistance, cellular senescence, and stem cell maintenance, offering insights into their contributions to lifespan regulation. Functional assays in these models, such as senescence-associated β-galactosidase staining and flow cytometry for apoptosis markers, could connect molecular mechanisms to organismal phenotypes. Integrating these experimental and computational approaches may deepen understanding of p53’s evolutionary role in lifespan regulation, uncovering novel therapeutic targets for age-related diseases and cancer.

The results of this study have broad implications for understanding the evolutionary and molecular underpinnings of longevity. By identifying RPLARs as potential modulators of p53’s regulation of longevity, this work advances the current understanding of how species-specific protein variations contribute to lifespan regulation. Our findings that RPLARs are concentrated in critical domains such as the DBD and PRD highlight their potential role in shaping p53’s interactions with DNA and co-regulators involved in apoptosis and cellular senescence. By bridging the gap between evolutionary biology and molecular mechanisms, this study provides a framework for leveraging cross-species insights into p53’s function to inform the development of longevity-enhancing therapies and novel cancer treatments.

## Materials and methods

### Observed average lifespan data, normalization, and protein sequences

Where available, the observed average lifespan for each organism was obtained from the Animal Diversity Web (https://animaldiversity.org/; accessed on 13 January 2024), the AnimalLifeExpectancy (a collation of lifespan data from AnAge, UMICH, Max Planck, PanTHERIA, Arkive, UKC, AKC) databases as well as from Smithsonian National Zoo Database. No average lifespan data could be found for more than two-thirds of the organisms in the 386-vertebrates dataset. However, maximum observed lifespan data were available for these species in the AnAge database. Thus, we normalized these maximum lifespan values to estimate the average lifespan values for our model construction. To achieve this, we first identified species for which both the maximum lifespan (L_max_) and average lifespan (L_avg_) were available and used this subset to train a simple linear regression model:


$${\text{L}}_{\text{avg}}\;\text{=}\;\;\;\;\alpha \;\;\;{\text{L}}_{\text{max}}\;\text{+}\;\;\;\;\beta \;\;\;$$

where α is the regression coefficient and β is the intercept. Using this model, we estimated missing L_avg _est_ values for species where only L_max_ was available. Following this, we computed a normalized lifespan (L_norm_) that accounts for the discrepancy between maximum and average lifespans using the learned α coefficient:


$${\text{L}}_{\text{norm}}\text{=}{\text{L}}_{\text{avg }\;\;\_\;\;\text{ est}}\text{+ (}{\text{L}}_{\text{max}}\text{-}{\text{L}}_{\text{avg }\;\;\_\;\;\text{ est}}\text{) }\;\;\alpha \;\;\;$$

This approach allowed us to derive a more representative lifespan measure across species while maintaining consistency with available empirical data. Python script of the normalization module can be found in S1 Protocol. Note that Normalized lifespan was only used in the 386-vertebrate model; raw average lifespan data (obtained from ADW and ALE) was used for the order-specific models.

Protein sequences in RES, SIFT, and PEPPI studies for all organisms were obtained from the UniProt database (https://www.uniprot.org/; last accessed on 09 September 2024) or the NIH’s GenBank database (https://www.ncbi.nlm.nih.gov/genbank/; last accessed 12 October 2023). For the 386-vertebrates dataset, the “all” ortholog dataset was obtained from GenBank under gene accession number 7157. See S2 File in the Supporting Information section for a list of the organisms, their observed average and normalized average lifespans, and UniProt or GenBank accession numbers for protein sequences used throughout the study.

### RES algorithm: Per residue evolutionary change quantification

Using the MUSCLE algorithm, p53 sequences were aligned by taxonomical order, and then the position weight matrix and background frequency values were calculated from each alignment. The position weight matrix is calculated by first counting the frequency of each amino acid (or gap) at every position in the alignment, then normalizing the count to the probability that a particular amino acid will be found at a particular position in the alignment. Included in this count of frequency is a pseudo-count to avoid a count of zero–an occurrence unlikely in naturally occurring protein sequences. We then calculated a corresponding dictionary of the overall (background) frequency of each amino acid (or gap) in the alignment. Including background frequency information in multiple sequence scoring algorithms improves scoring and identification of functional sites. Following this, we used our position weight matrix and background frequencies to score each position of individual aligned sequences by taking the log odds ratio of observed frequency to background frequency. Log-odds scoring has been used in most pair-wise scoring, as it formalizes differentiating between more functional and less functional positions [38]. We used the MUSCLE default gap opening and extension penalties of -2.90 and 0, respectively in our scoring.

### Phylogenetic generalized least squares and statistical significance analyses

The PGLS analysis was used to account for the non-independence in the evolution of p53 orthologs due to shared evolutionary history and to provide statistical significance testing for the RES-average lifespan model [10]. Assuming that the evolution of p53 and average lifespan occurred at constant rates and in random directions, we used the Brownian motion model to describe the covariance between the p53 sequence and average lifespan. We used *nlme*, *ape*, *seqinr*, *Biostrings*, *phytools*, and *phangorn* packages in R to conduct these analyses. We first used ape to construct phylogenetic trees of the p53 orthologs from each of the six MUSCLE alignments. Next, we used the corBrownian method to create a Brownian motion model of p53 ortholog phylogenetic relationships. We then used the *nlme* package to calculate the r and p-values between average lifespan (response variable) and RES (predictor variable) for all positions of the aligned sequences. Full source code for the RES and PGLS workflows can be accessed in the S1 Protocol in the Supporting Information section. To assess the phylogenetic signal between each p53 alignment and average lifespan, we used the *phylosig()* method in R. Additionally, the *AIC()* and *logLik()* methods were used to characterize the model fitting for each of the seven datasets.

### SIFT: Functional impact of RPLAR mutation on p53

To assess the functional impact of RPLAR mutation on p53 function, we employed SIFT, which employs a sequence homology-based algorithm to distinguish between tolerant and intolerant protein mutations [39]. We compared missense mutations of p53 sequences belonging to the longest and shortest-lived organisms. If the two sequences had the same residue at a particular point, the organism with the next shortest lifespan and a residual change was used in the prediction. Missense mutations in the ortholog of (one of the) shortest-lived organisms in each order are determined from MEGA alignment.

### p53 cancer hotspot and transactivation enrichment analyses

The results of Kato’s yeast functional assay study of over 2000 mutant human p53 transactivation of key genes involved in its mechanisms of tumor suppression are cataloged in the National Cancer Institute’s *TP53* database (https://tp53.cancer.gov; last accessed Dec 09, 2024). We used this data to assess the effect of RPLAR-specific mutants on transactivation. We aligned RPLARs across all orders to the human p53 ortholog, then created mutants in the form XnY, where X is the wild-type human p53 residue at position n and Y is the mutant residue belonging to the species with the shortest or longest average lifespan in the alignment from which it was originally mapped. We then queried the Functional/Structural Data by Gene Variant. Next, we searched the data by Protein Description, batch entering the names of the RPLAR mutants and selecting the first entry’s result. Finally, we recorded transactivation data for *MDM2*, *NOXA*, *WAF1*, *AIP1, WAF1, H1433s, and GADD45* from the table titled “Systematic Assessment of Transactivation Capacities in Yeast and Saos2 Assays by Kato (2003) and Kakudo (2005)” [15]. In addition to the results of Kato’s study, the results of Chang et al. cancer hotspot studies are cataloged in the *TP53* database (accessed on January 30, 2025: https://tp53.cancer.gov/). All 30 HSS mutants were enriched with these results to determine if the HS or HL mutant constituted a cancer hotspot. To further assess the pathogenicity of the mutants, the ClinVar database was manually searched for *TP53-*specific data (accessed on August 12, 2024; https://www.ncbi.nlm.nih.gov/clinvar) [40,41].

### Rosetta Cartddg2020 protocol

Rosetta was used to assess the free energy change of p53 folding following the introduction of single residue mutations at the RPLARs in an AlphaFold3-predicted model of wild-type human p53. For conformational sampling, Rosetta employs a step-wise approach where it first locates many local minima in the coarse-grained low-resolution potential, then it searches for the lowest free-energy minimum and refines the coarse-grained structure by adding back missing atomic details. Finally, to optimize the geometry of the minimum energy structure, a step-wise Monte Carlo minimization is carried out. In this step, a random assortment of torsion angles is perturbed. This is followed by rotamer optimization and then continuous side chain and backbone gradient descent minimization. Energy functions in Rosetta capture the thermodynamic properties of protein folding with high accuracy. The packing created by atom-atom interactions is calculated using Lennard-Jones potential; the implicit solvent model describes the dominant hydrophobic effect; an electrostatic desolvation cost quantifies the effect of burying polar atoms; hydrogen bonding is described by an explicit hydrogen-bonding potential [42,43].

Following p53 monomer predicted in AlphaFold3, the Cartesian Free Energy (Cartddg2020) Rosetta package was used to mutate p53 via alanine substitution and assess free energy change. Cartddg2020 first relaxes the wild-type structure in Cartesian space using the FastRelax protocol. It then samples conformations to determine the best structure for the wild-type and each mutant. Following this, it carries out a second round of FastRelax in Cartesian space, only allowing movement of the backbone within 3 residues and sidechains that are within 6Å of the mutated residue. The change in free energy between the optimized wild-type structure and the optimized mutant structure is calculated by multiplying their energy gaps and the scaling factor of the energy function-specific scaling factor [44,45]. The talaris2014 scoring energy function was used to assess mutational free energy [46]. A total of 108 single residue mutations were tested on p53: the 30 RPLARs mapped to the human ortholog, a total of 63 non-RPLARs comprising those residues within a two-residue window of each RPLAR, and the 15 cancer hotspot mutations, including six major hotspots.

### PEPPI: Predicting interactome of p53/p21 cellular senescence pathways

PEPPI, Pipeline for the Extraction of Predicted Protein-Protein Interactions, is a computational pipeline designed to predict protein-protein interactions (PPIs) by assessing the likelihood of direct physical interactions between pairs of query sequences. The tool combines five independent prediction modules: structural homology generated from monomeric protein sequence threading, sequence homology (using SEQ), the conjoint-triad neural network-based approach, functional association data (STRING), and a negative interaction filter (SPRINGNEG). The likelihood of interaction is quantified as a natural log-transformed likelihood ratio (log(LR)), determined through a consensus of these modules. The consensus is derived using a naïve Bayesian classifier model, trained on 800 high-confidence interactions and 800 curated non-interactions from experimentally and network-validated databases. PEPPI calculates the likelihood of interactions as follows:


$$log(LR)\;=\;\sum_{i=1}^{n}\log(p(x_{i}|I))\;-\;\log(p(x_{i}|NI))$$

where p(x_i_ | I) and p(x_i_ | NI) denote the conditional probabilities from the i-th prediction module in the interacting and non-interacting reference distributions, respectively [47].

In this study, PEPPI was employed to construct and compare micro p53/p21 senescence interactomes for Primates and Perciformes. It predicted the likelihood of interactions between p53 and several proteins known to be involved in p53-mediation of the p21 cellular senescence pathway in mice and human p53, including Smad2, Smad3, Npm1, MDM2, and Klf4. Additionally, interactions between MDM2 and Rpl11, as well as between Pras40 and Akt of the Primates and Perciformes orthologs, were assessed. PEPPI requires as input the fasta format of the two protein sequences whose interaction is to be predicted. For all the aforementioned interactions and 8–10 species in the orders Primates and Perciformes, we obtained the protein sequence for each organism from UniProt. We then utilized the PEPPI webserver for input and output processing (https://zhanggroup.org/PEPPI; last accessed July 2024).

### AlphaFold and PyMOL: Assessing the involvement of RPLARs in PEPPI predicted interactions

We used AlphaFold to predict the structures of p53-Smad2, p53-Smad3, p53-Klf3, and p53-Nmp1 complexes for organisms with a noticeably higher likelihood of interactions based on the heatmap of PEPPI results. We then used the interfaceResidues PyMol module to predict and visualize the interacting residues between all predicted complexes (https://pymolwiki.org/index.php/InterfaceResidues; last accessed Dec 15, 2024). This module calculates interface residues by taking the difference between the area of a protein complex and the area of the complex’s monomers. Residues whose difference in accessible surface area from the complex to a single chain is greater than this cutoff are considered interface residues. We used a cutoff of 2 Å to determine p53 interface residues [48].

## Supporting information

S1 Fig Pymol visualization of interface residues in AlphaFold predicted p53-mediated cellular senescence complexes. (A) p53-Klf4 (*H. sapiens*). (B) p53-Nmp1 (*S. boliviensis*). (C) p53-Smad2 (*L. bergylta*). (D) p53-Smad2 (*P. troglodytes*). (E) p53-Smad3 (*L. bergylta*). (F) p53-Smad3 (*P. troglodytes*). In all images, p53 is green and its interface residues are labeled with one-letter code and are in purple. The second binding monomer is in cyan.
(TIF)

S1 File RPLAR r, p-value, SIFT score data for order-specific and 386-vertebrate models. (PDF)

S2 File Protein accession numbers and average lifespan data of all organisms in RES and PEPPI studies. (PDF)

S3 File Results from PEPPI prediction of p53-mediated cellular senescence protein-protein interactions. (PDF)

S1 Protocol Zip file containing source code for RES and PGLS analysis. The code is also accessible in the author’s GitHub repository.
(ZIP)
