# Supplementary figures and images for: Structural and mechanistic diversity in p53-mediated regulation of organismal longevity across taxonomical orders
Source: PLoS Comput Biol. 2025 May 2;21(5):e1012382. doi: 10.1371/journal.pcbi.1012382 (PMC12068700; doi:10.1371/journal.pcbi.1012382)

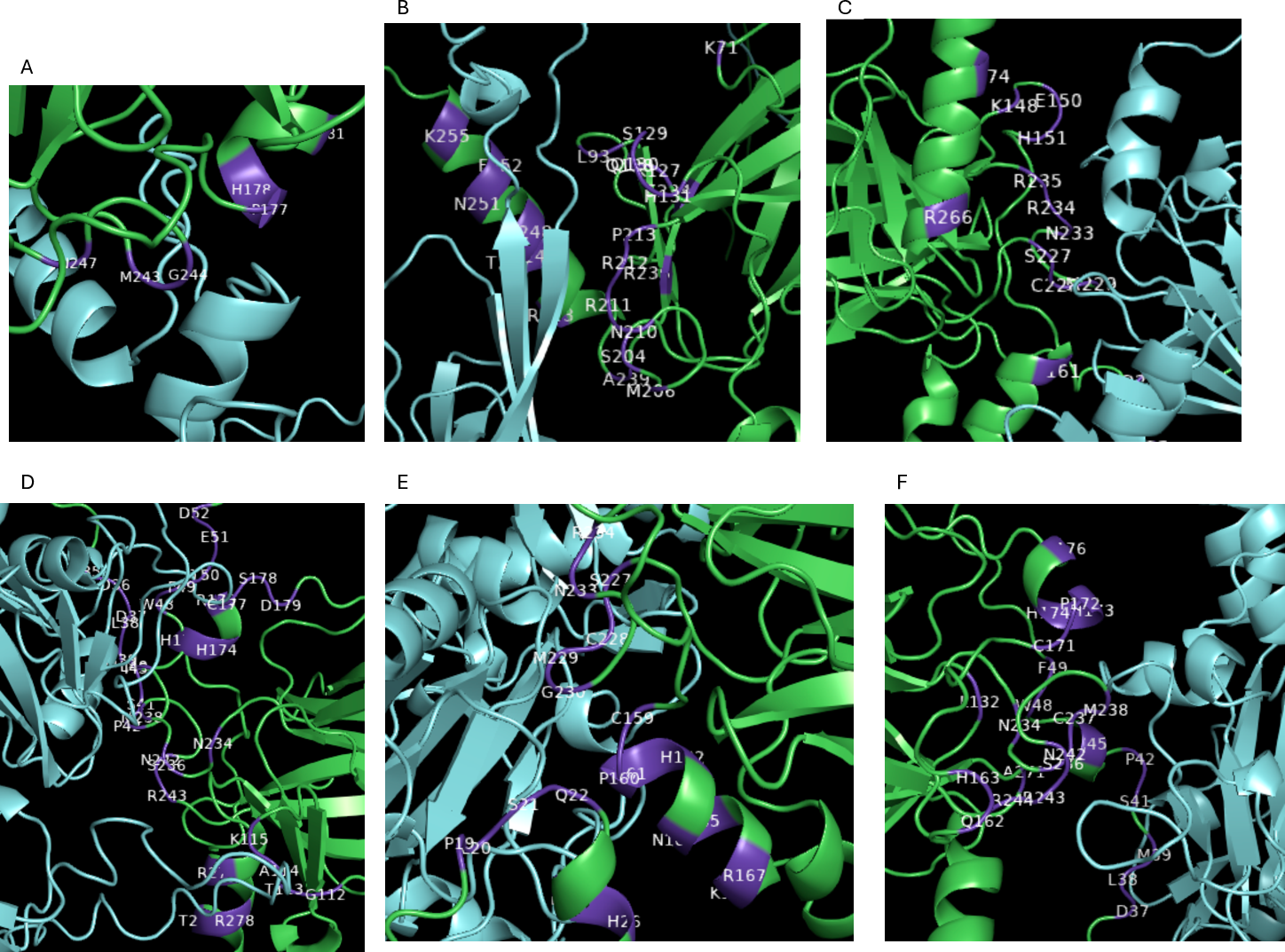

Supplement: S1 Fig — (A) p53-Klf4 (H. sapiens). (B) p53-Nmp1 (S. boliviensis). (C) p53-Smad2 (L. bergylta). (D) p53-Smad2 (P. troglodytes). (E) p53-Smad3 (L. bergylta). (F) p53-Smad3 (P. troglodytes). In all images, p53 is green and its interface residues are labeled with one-letter code and are in purple. The second binding monomer is in cyan. (TIF) [file pcbi.1012382.s001.tif]
