# Supplementary material for: Structural and mechanistic diversity in p53-mediated regulation of organismal longevity across taxonomical orders
Source: PLoS Comput Biol. 2025 May 2;21(5):e1012382. doi: 10.1371/journal.pcbi.1012382 (PMC12068700; doi:10.1371/journal.pcbi.1012382)
Supplement: S1 File — (PDF) [file pcbi.1012382.s002.pdf]

Order-specific models

Organism	RPLAR	Coefficient, r	p-value	Longest to shortest mutant	SIFT score
H. sapiens	V10	-5.826088	0.03151946	V10I	1
	Q104	-5.66002	0.03873592	Q104H	0.06
D. eleginoides	A73	-3.802489	0.0167092353	A73N	0.59
	P78	-9.13091	0.010784275	P78M	0.01
	D81	-10.444539	0.0115399996	D81E	1
	M82	3.802428	0.022792765	M82T	0.49
	S99	-7.878121	0.0035875974	S99T	0.85
	Q110	-12.272993	0.0004243929	Q110R	0.77
	T114	-11.404453	0.0004243929	T114S	1
	L145	-6.869107	0.0127471376	L145V	1
	L151	-5.019179	0.023195375	L151Q	0.61
	D184	-10.444539	0.0115399996	D184E	1
	N205	-5.871277	0.0303530404	N205H	0.64
	L217	-7.051887	0.0021475204	L217H	0.01
	M287	3.737583	0.0208098261	M287S	0.73
	R312	-11.424809	0.0004243929	R312K	1
	T316	-4.237944	0.0201956261	T316A	0.79
	R359	-5.79518	0.0235943946	R359K	1
	K381	-5.244161	0.0427700948	K381R	0.68
H. glaber	E71	-7.043893	5.93E-03	E71Q	0.69
	R172	-4.407609	1.63E-02	R172K	0.36
	D219	-8.212534	5.61E-05	D219E	1
	L220	-3.629635	1.15E-02	L220P	1
	H288	-4.513572	4.78E-02	H288R	1
	G309	3.327386	3.60E-02	G309C	0.08
B. phylasus	E60	8.715124	0.035049694	E60D	0.28
	P62	7.317099	0.0465509	P62S	0.69
	M64	5.305372	0.034750596	M64V	0.87
	R103	61.65156	0.009095075	R103H	0.2
	H204	-10.295093	0.036513837	H204N	0.7
	S255	-7.879708	0.044779536	S255R	0.39
	G290	-10.783718	0.002598738	G290C	0.18
	S305	44.987832	0.00581809	S305G	0.34
H. sapiens (cross-order)	G112	-13.109216	0.001111743	G112R	0.59
	H115	-7.907238	0.00438665	H115K	0.37
	A129	16.777108	0.015132165	A129S	0.74
	N311	41.952691	0.049929212	N311G	0.41
	Q331	15.232961	0.01714413	Q331K	0.63
	Q356	18.058757	0.017454934	G356E	1

386-vertebrate model

With gap position	Coefficient	p-value	Residue of longest-lived (C. abingdonii)	Residue of shortest-lived (O.melastigma)	Residue of H. sapiens
resi_118	-2.334147	3.44E-04	A118	M1	M1
resi_119	-2.377649	6.89E-03	G119	-	E2
resi_123	-2.472658	1.22E-02	M123	V4	5Q
resi_124	-2.516817	2.72E-02	L124	S5	S6
resi_133	-2.164415	1.12E-02	D133	D6	D7
resi_134	-3.484023	1.32E-04	P126	V7	P8
resi_140	-2.43532	3.23E-02	G127	-	S9
resi_141	-3.969577	8.28E-06	L128	-	V10
resi_166	-1.784451	3.95E-02	S141	S10	S15
resi_167	-2.746746	1.23E-04	Q142	Q11	Q16
resi_174	-2.364018	2.27E-02	S144	S13	T18
resi_177	-3.019479	2.54E-04	D147	E16	D21
resi_232	-1.685262	2.94E-02	R155	V21	N30
resi_233	-2.869993	3.58E-03	T156	C22	V31
resi_267	6.531168	2.74E-05	-	-	P34
resi_273	3.606813	2.19E-02	Q158	-	L35
resi_283	-1.887385	1.35E-02	L168	L26	M40
resi_302	2.741557	4.07E-04	P174	N28	P47
resi_308	3.355312	1.62E-02	-	-	D48
resi_320	-2.619541	3.27E-02	S177	P30	E51
resi_322	-4.377303	1.02E-07	-	S33	Q52

resi_331	-2.21662	2.74E-02	L178	L35	F54
resi_332	2.266809	9.24E-03	D179	P36	T55
resi_347	2.917504	9.56E-03	L182	-	P58
resi_349	2.771322	2.18E-03	D184	P37	P60
resi_352	2.964547	2.21E-03	D187	M40	A63
resi_353	3.39528	1.51E-03	P188	L41	P64
resi_354	-3.55531	2.88E-03	S189	P42	R65
resi_410	3.190065	4.12E-03	L191	G44	A70
resi_411	2.609983	1.19E-02	L192	E45	P71
resi_420	3.333337	4.34E-03	D199	D53	A74
resi_440	2.069864	4.35E-03	P205	P59	P80
resi_441	-2.233092	3.29E-02	D206	L60	T81
resi_444	-2.415575	2.27E-02	P209	I63	A84
resi_445	2.475184	6.44E-03	E210	A64	P85
resi_446	3.336716	7.66E-04	P211	I65	A86
resi_460	-3.662019	8.89E-03	-	-	S90
resi_464	-4.937313	2.13E-06	-	N69	W91
resi_503	2.509151	3.84E-02	S222	V77	S99
resi_504	1.913315	4.75E-02	T221	T78	Q100
resi_506	3.638613	2.07E-04	D223	D80	T102
resi_508	-3.440653	1.85E-03	A225	P82	Q104
resi_515	-2.478192	4.56E-02	L232	L89	L111
resi_520	-3.44557	5.87E-03	S237	S94	S116
resi_527	-5.583113	1.19E-05	T244	T101	T123
resi_533	-4.62473	5.59E-03	Q250	T107	A129
resi_537	-8.850565	5.01E-04	L254	L111	M133
resi_540	-7.846415	3.91E-06	Q257	Q114	Q136
resi_544	-8.471128	2.22E-08	T261	T118	T140
resi_553	-3.121572	5.97E-03	S270	R127	S149
resi_557	2.261049	2.31E-02	A274	K131	P153
resi_559	-5.628363	2.21E-03	S276	A133	T155
resi_560	-4.147748	5.86E-03	I277	V134	R156
resi_563	3.535401	3.49E-02	A280	A137	A159
resi_564	-3.439517	8.69E-03	T281	T138	M160
resi_566	-7.20941	3.34E-02	V283	V140	I162
resi_572	4.980742	1.55E-03	H289	D156	H168
resi_576	5.634984	5.08E-03	V293	V160	V172
resi_578	4.002638	1.48E-02	R295	R162	R174
resi_612	-2.446355	2.89E-02	Q324	L177	R202
resi_613	-3.687437	1.37E-04	A325	A178	V203
resi_619	4.502005	7.26E-05	E331	P184	R209
resi_627	-3.144821	1.52E-02	T339	T192	V217
resi_632	2.597949	6.53E-03	T344	P197	P222
resi_635	-3.788838	5.31E-04	L347	P200	V225
resi_639	-2.713605	1.33E-03	C351	M204	C229
resi_643	-9.650987	5.03E-05	L355	L208	H233
resi_645	-5.330846	2.77E-06	N357	S210	N235
resi_662	4.420821	5.20E-04	L374	L227	L252
resi_663	-4.922009	7.37E-04	A375	T228	T253
resi_665	-2.651899	2.91E-02	I377	L230	I255
resi_666	6.874481	2.61E-04	T378	T231	T256
resi_669	-5.328305	2.29E-04	G381	T234	D259
resi_672	-7.461722	6.79E-07	H383	A237	S261
resi_686	-6.301183	2.75E-04	R384	G251	G262
resi_693	3.478228	3.79E-02	C391	C258	S269
resi_707	-7.090248	2.54E-02	R405	K272	R283
resi_711	2.260112	1.86E-02	E409	E276	E287
resi_713	-3.513346	1.40E-07	F411	R278	L289
resi_725	-4.133991	5.62E-04	G417	-	P295
resi_726	-3.245271	5.56E-04	R418	-	H296
resi_735	-2.084295	4.43E-02	L420	T282	E298
resi_736	-2.511714	2.82E-02	N421	Q283	L299
resi_747	-2.418426	3.68E-02	G422	P284	P300
resi_807	-2.19446	1.68E-02	Q436	T290	P309
resi_808	-3.948178	2.40E-03	A436	P291	N310
resi_819	-3.81935	1.85E-04	T441	S294	S314

resi_820	-2.487591	1.12E-02	A442	S295	S315
resi_822	-3.360409	3.30E-03	N444	K297	Q317
resi_833	2.334286	4.73E-02	V450	S303	L323
resi_851	-5.721272	3.27E-06	K454	K310	G325
resi_858	-2.30118	3.28E-02	V456	V312	Y327
resi_860	-3.025646	2.09E-03	L458	H314	T329
resi_862	2.128176	1.05E-02	E460	R316	Q331
resi_872	2.864018	4.07E-03	M468	E324	E339
resi_875	4.947847	2.11E-05	K471	K327	R342
resi_891	-5.068199	8.72E-08	K483	E339	Q354
resi_894	-4.733871	8.80E-08	G486	K342	K357
resi_897	-3.163542	4.67E-03	P488	P344	P359
resi_900	-2.123951	3.38E-02	H491	I347	S362
resi_901	2.605534	4.28E-03	R492	S349	A364
resi_902	3.102306	5.80E-03	N493	S350	H365
resi_949	-4.041256	3.39E-03	T495	-	S367
resi_953	-3.664822	5.28E-05	L499	-	L369
resi_957	-2.304172	2.67E-02	R503	-	K370
resi_958	-6.075223	4.10E-07	K504	-	S371
resi_970	-2.464067	3.51E-03	S506	-	K373
resi_985	-2.198375	3.53E-02	V510	-	T377
resi_993	3.905019	3.86E-03	G515	-	-
resi_1025	-2.765761	1.32E-02	E522	-	T387
resi_1026	-2.773155	3.88E-04	E523	-	E388
resi_1029	-3.979126	1.08E-03	-	T358	P390

- = predicted gap
