# Supplementary material for: Structural and mechanistic diversity in p53-mediated regulation of organismal longevity across taxonomical orders
Source: PLoS Comput Biol. 2025 May 2;21(5):e1012382. doi: 10.1371/journal.pcbi.1012382 (PMC12068700; doi:10.1371/journal.pcbi.1012382)
Supplement: S2 File — (PDF) [file pcbi.1012382.s003.pdf]

Order: Perciformes

Organism			Sequence accession numbers								
Scientific name	Common name	Average lifespan (years)	p53	Smad2	Smad3	Npm1	Rpl11	Mdm2	Pras40	Akt	Klf4
Thunnus albacares	Yellowfin tuna	9	XP_044196743.1	UPI001C4B2943	UPI00054C1478	/	/	/	/	/	/
Perca flavescens	Yellow perch	12	A0A484CCM0	UPI00106EAF7A	A0A484DP09	A0A484CM55	A0A484CRN0	A0A484BZ23	A0A6A5ENB6	A0A484C4T4	A0A484CJE2
*Dicentrarchus labrax**	European seabass	15	A0A8C4EKX1	A0A8C4E8G4	A0A8C4F4T0	A0A8C4I739	UPI0021F50FAC	A0A8C4DZ38	A0A8C4DEU5	A0A8C4DG29	A0A8C4HU78
Platichthys flesus	European flounder	15	O12946	UPI002DB75018	/	/	/	/	/	/	/
Sander lucioperca	Zander	16	A0A8C9ZEJ7	A0A8D0D734	A0A8C9ZTJ9	A0A8C9XKY4	A0A8D0AAC8	A0A8C9XZT3	A0A8C9YHW4	A0A8C9XJN6	A0A8C9XNT1
Scomber scombrus	Atlantic mackere	17	UPI002DD90AFB	UPI002DD915F3	/	/	/	/	/	/	/
Perca fluviatilis	European perch	22	A0A6A5F0Y3	A0A6A5EE76	A0A6A5FEG0	A0A6A5ESY6	A0A6A5ELS6	A0A6A5DZX5	/	A0A6A5DQU5	A0A6A5EH34
Epinephelus coioides	Orange-spotted groupe	22	F8RKR1	/	/	/	/	/	/	/	/
Labrus bergylta	Ballan wrasse	29	A0A3Q3GRI3	UPI000F31D7C5	A0A3Q3MUV6	A0A3Q3LSC0	UPI000F30F9F7	A0A3Q3FQT2	A0A3Q3E1S6	A0A3Q3GN09	A0A3Q3G1D1
Anabas testudineus	Climbing perch	7	A0A3Q1JCZ8	A0A7N6BK16	A0A3Q1J1S9	A0A7N6AAM7	A0A3Q1IK23	A0A3Q1JLB4	A0A3Q1JP30	UPI00194B08E9	A0A3Q1J2Y9
*Amphiprion percula**	Orange clownfish	18	A0A3P8T0X9	A0A3P8RXC7	UPI000F387D83	A0A3P8SIS1	A0A3P8TH83	A0A3P8TU83	A0A3P8TXZ2	A0A3P8RL25	A0A3P8TF24
Sparus aurata	Gilt-head bream	11	A0A671VUB9	A0A671XQA2	A0A671TKP9	A0A671TUQ4	A0A671YTZ8	A0A671Y7M9	A0A671XJ86	A0A671XJ74	A0A671VEU0
Acanthochromis polyacanthus	Spiny chromis	6	A0A3Q1HGV2	/	/	/	/	/	/	/	/
Parambassis ranga	Indian Glass Fish	5.5	A0A6P7K8V8	/	/	/	/	/	/	/	/
Sphaeramia orbicularis	Orbiculate cardinalfish	4	A0A673CZK1	/	/	/	/	/	/	/	/
Salarias fasciatus	Lawnmower blenny	3	A0A672G194	/	/	/	/	/	/	/	/
Thunnus maccoyii	Southern bluefin tuna	20	UniRef100_UPI001C4B3AFC	/	/	/	/	/	/	/	/
Amphiprion ocellaris	Ocellaris clownfish	7	A0A3Q1AKW0	/	/	/	/	/	/	/	/
Lates calcarifer	Barramundi	20	A0A4W6CU25	/	/	/	/	/	/	/	/
Etheostoma spectabile	Orangethroat darter	4	A0A5J5CEU8	/	/	/	/	/	/	/	/
Channa striata	Snakehead murrel	10	A0AA88NQU6	/	/	/	/	/	/	/	/
Dissostichus eleginoides	Patagonian toothfish	50	A0AAD9F682	/	/	/	/	/	/	/	/
Dicentrarchus labrax	European seabass	15	A0A8C4EKX1	/	/	/	/	/	/	/	/
Amphiprion percula	Orange clownfish	18	A0A3P8T0X9	/	/	/	/	/	/	/	/

Order: Primates

Organism			Protein sequence accession numbers								
Scientific name	Common name	Average lifespan (years)	p53	Smad2	Smad3	Npm1	Rpl11	Mdm2	Pras40	Akt	Klf4
Callithrix jacchus	Common marmoset	10	A0A2R8MYD9	/	/	/	/	/	/	/	/
Carlito syrichta	Philippine tarsier	13	A0A1U7U5H4	/	/	/	/	/	/	/	/
Microcebus murinus	Gray mouse lemur	14	A0A8B7H7E6	A0A8B7F143	A0A8C5YBL9	A0A8C5YAS8	A0A8C5XZX6	A0A8C5V8R7	A0A8C6EKZ4	A0A8C5VV71	A0A8B7EFL3
Mandrillus leucophaeus	Drill	28	A0A2K6AAH4	/	/	/	/	/	/	/	/
Macaca fuscata fuscata	Japanese macaque	30	P61260	/	/	/	/	/	/	/	/
Sapajus apella	Tufted capuchin	40	A0A6J3I233	A0A6J3GBM2	A0A6J3F4R4	A0A6J3GDU9	A0A6J3JQA4	A0A6J3IMI1	A0A6J3GQB8	A0A6J3GYY1	A0A6J3EU19
*Gorilla gorilla gorilla**	Gorilla	47	G3R2U9	G3QPV6	A0A2I2ZDG8	A0A2I2YQY6	G3RWD2	A0A2I2ZWM5	G3S586	G3RB32	G3SJ37
Pan troglodytes	Chimpanzee	56	A0A2I3S1V8	H2QEH9	A0A2I3TN10	A0A2J8LXX4	A0A2I3TKT2	H2R792	K7AMC0	K7AGW5	H2QXN2
Homo sapiens	Human	76	P04637	Q15796	P84022	P06748	P62913	Q00987	Q96B36	P31749	O43474
Macaca mulatta	Indochinese rhesus macaque	26	P56424	F7CP27	A0A1D5Q1C9	F7AWJ2	F6PJ30	G7N7L5	A0A5F8AIB7	G7MWI7	A0A5F8AGZ8
Macaca fascicularis	Crab-eating macaque	38	P56423	/	/	/	/	/	/	/	/
Pan paniscus	Bonobo	41.5	A0A2R9A5P4	/	/	/	/	/	/	/	/
*Saimiri boliviensis boliviensis**	Black-capped squirrel monkey	17.5	A0A2K6SLC5	A0A2K6V827	A0A2K6SKP9	A0A2K6UT13	A0A2K6V210	A0A2K6S797	A0A2K6UND0	A0A2K6SGS8	A0A2K6SJ96
*Theropithecus gelada**	Gelada	20.8	A0A8D2F964	A0A8D2GAI2	A0A8D2G920	A0A8D2K0P5	A0A8D2EEE2	A0A8D2K6W8	A0A8D2E2Q4	A0A8D2F2J1	A0A8D2E5U5
Rhinopithecus bieti	Black-and-white snub-nosed monkey	23	A0A2K6LYM5	A0A2K6KLL7	A0A2K6K5G6	A0A2K6MQI9	A0A2K6K899	A0A2K6KDN6	A0A2K6KJD1	A0A2K6KHG5	A0A2K6JXF0
*Papio anubis**	Olive baboon	25.2	A0A096P2R8	A0A096NP77	A0A096NMF6	A0A2I3MVB3	UPI000440407B	A0A2I3NF10	A0A0A0MU05	A0A2I3N0A6	A0A8I5R2U0

Cebus imitator	Panamanian white-faced capuchin	45	A0A2K5RMZ8	/	/	/	/	/	/	/	/
Pongo abelii	Sumatran orangutan	55	A0A2J8RWD8	/	/	/	/	/	/	/	/
Chlorocebus sabaeus	Green monkey	12	A0A0D9RG12	/	/	/	/	/	/	/	/
Colobus angolensis palliatus	Angolan colobus	35.3	A0A2K5K834	/	/	/	/	/	/	/	/
Cercocebus atys	Sooty mangabey	26.8	A0A2K5N3Z0	/	/	/	/	/	/	/	/
Propithecus coquereli	Coquerel's sifaka	28	A0A2K6GZU4	/	/	/	/	/	/	/	/
Rhinopithecus roxellana	Golden snub-nosed monkey	29.5	A0A2K6QDI6	/	/	/	/	/	/	/	/
Prolemur simus	Greater bamboo lemur	17.6	A0A8C9A948	/	/	/	/	/	/	/	/
Piliocolobus tephrosceles	Ugandan red colobus	12.1	A0A8C9GX67	/	/	/	/	/	/	/	/
Nomascus leucogenys	Northern white-cheeked gibbon	44.1	G1RF61	/	/	/	/	/	/	/	/
Otolemur garnettii	Garnett's greater galago	20	H0XGB0	/	/	/	/	/	/	/	/
Theropithecus gelada	Gelada	20.8	A0A8D2F964	/	/	/	/	/	/	/	/
Papio anubis	Olive baboon	25.2	A0A096P2R8	/	/	/	/	/	/	/	/

Order: Rodentia

Organism			Sequence accession numbers
Scientific name	Common name	Average lifespan (years)	p53
Mus musculus	House mouse	2	P02340
Cricetulus griseus	Chinese hamster	2.5	O09185
Mesocricetus auratus	Golden hamster	2.5	Q00366
Cavia porcellus	Guinea pig	5	Q9WUR6
Octodon degus	Common degu	6.5	A0A6P3F490
Chinchilla lanigera	Long-tailed chinchilla	10	A0A8C2YMX2
Castor canadensis	American beaver	11	A0A250YHC8
Spalax judaei	Middle East blind mole-rat	21	Q68VB0
Heterocephalus glaber	Naked mole-rat	31	G5B5D6
Mastomys natalensis	Natal multimammate mouse	0.25	P89002
Rattus norvegicus	Brown rat	3.8	P10361
Peromyscus maniculatus	Deer Mouse	8.7	A0A8C8TJT9
Meriones unguiculatus	Mongolian gerbil	6.3	Q920Y0
Ictidomys tridecemlineatus	Thirteen-lined ground squirrel	7.9	I3N5N2
Marmota monax	Groundhog	14	O36006
Microtus ochrogaster	Prairie vole	5.3	A0A172Q425
Dipodomys ordii	Ord's kangaroo rat	9.9	A0A1S3EYG2
Eospalax fontanierii baileyi	Plateau zokor	4.5	A0A1S6QMR1
Mastomys coucha	Southern multimammate mouse	3	A0A1U9W5F4
Marmota marmota marmota	Alpine marmot	17.4	A0A8C6A0W6
Mus spicilegus	Mound-building mouse	3.5	A0A8C6I657
Urocitellus parryii	Arctic ground squirrel	9	A0A8D2I1W0
Microtus duodecimcostatus	Mediterranean pine vole	2.5	A0A8K1Z883
Otospermophilus beecheyi	California ground squirrel	8	Q64662

Order: Artiodactyla

Organism			Sequence accession number
Scientific name	Common name	Average lifespan (years)	p53
Lipotes vexillifer	Baiji	24	A0A340X8E6
Delphinapterus leucas	Beluga whale	40	Q8SPZ3
Tursiops truncatus	Common bottlenose dolphin	45	A0A2U4C2U9
Monodon monoceros	Narwhal	50	A0A4V5P9N3
Globicephala melas	Long-finned pilot whale	60	UPI00293D9E1D
Eubalaena glacialis	North Atlantic right whale	67	UPI002A5A2F50
Physeter macrocephalus	Sperm whale	68	A0A455C1G1
Balaenoptera musculus	Blue whale	85	A0A8C0DFA9
Balaenoptera physalus	Fin whale	114	A0A6A1Q3Q7
Balaenoptera acutorostrata	Common minke whale	57	A0A383YUA7

Lagenorhynchus albirostris	White-beaked dolphin	40	XP_059988824.1
Mesoplodon densirostris	Blainville's beaked whale	27	XP_059937197.1
Delphinus delphis	Short-beaked common dolphin	25	XP_059854544.1
Kogia breviceps	Pygmy sperm whale	17	XP_058904949.1
Phocoena phocoena	Harbour porpoise	13	XP_065753055.1
Ovis aries	Sheep	11	P51664
Sus scrofa	Wild boar	12	Q9TUB2
Muntiacus muntjak	Southern red muntjac	17	A0A5N3WLG9
Bos taurus	Cattle	20	P67939
Capra hircus	Goat	20.8	A0A452G0A3
Odocoileus virginianus texanus	Texas white-tailed deer	23	A0A6J0VGP1
Vicugna pacos	Alpaca	25	A0A6I9IU1
Bison bison bison	Plains bison	33.5	A0A6P3HQA5
Camelus bactrianus	Bactrian camel	35	A0A9W3H045
Hippopotamus amphibius kiboko	Hippopotamus	61	XP_057572051.1
Camelus dromedarius	Dromedary	45	A0A5N4D2L6
Bubalus bubalis	Domestic water buffalo	34.9	F6MDM8
Bos mutus grunniens	Wild yak	26.3	A0A0N7FDT7
Neophocaena asiaeorientalis asiaeorientalis	Yangtze finless porpoise	21.5	A0A341BQX3
Bos indicus	Zebu	20	P67938

Order: Carnivora

Organism			Sequence accession numbers
Scientific name	Common name	Average lifespan (years)	p53
Neovison vison	American mink	11.4	A0A8C7A9R2
Vulpes vulpes	Red fox	12	A0A3Q7TIN0
Canis lupus dingo	Dingo	14	A0A8C0K1L6
Nyctereutes procyonoides	Common raccoon dog	15.6	A0A811ZM26
Canis lupus familiaris	Dog	20.6	Q29537
Neomonachus schauinslandi	Hawaiian monk seal	25	A0A2Y9HEV7
Callorhinus ursinus	Northern fur seal	25	A0A3Q7NG00
Panthera leo	Lion	28	A0A8C8Y413
Felis catus	Cat	30	P41685
Ursus maritimus	Polar bear	38.2	A0A384BVC2
Zalophus californianus	California sea lion	30	A0A6J2FNY3
Lynx rufus	Bobcat	32.3	UPI001F1287F1
Enhydra lutris	Sea otter	19	A0A2Y9L4Q2
Panthera pardus	Leopard	22	A0A9V1DWE6
Suricata suricatta	Meerkat	12.5	A0A673UVH2
Odobenus rosmarus	Walrus	30	A0A2U3VT95
Leptonychotes weddellii	Weddell seal	25	A0A2U3Y5G3
Ursus americanus	American black bear	32	A0A452QA33
Lynx canadensis	Canada lynx	26	A0A667GWT3
Crocuta crocuta	Spotted hyena	41	A0A6G1A9W5
Acinonyx jubatus	Cheetah	17	A0A6J1YF15
Puma concolor	Cougar (Mountain lion)	20	A0A6P6HL51
Panthera tigris altaica	Siberian tiger	23	A0A8C9K4J3
Ailuropoda melanoleuca	Giant panda	30	G1MEP6
Mustela putorius furo	Domestic ferret	8	M3YC88

* indicates ortholog that are not used in RES alignments; they are instead used in PEPPI studies

/ not used in PEPPI studies due to limited sequence data

Dataset: Vertebrates (386 organisms)

Organism Name	Maximum longevity (years)	Average longevity (years)	Normalized lifespan (years)
H.sapiens	122.5	76	112.9855336
M.musculus	4	2	3.590775639
R.norvegicus	3.8	3.8	3.8
D.rerio	5.5	6.450855027	5.69455652
B.taurus	20	20	20
G.gallus	30	25.93785661	29.16883598
S.scrofa	27	12	23.93081729
C.lupus	20.6	18.4612111	20.16237774
O.aries	22.8	11	20.38557627
P.troglodytes	68	56	65.54465383
F.catus	30	30	30
M.domestica	5.1	6.1326999	5.311302978
M.mulatta	40	26	37.13542947
O.cuniculus	13	12.41626367	12.88056044
O.latipes	5	6.053161118	5.215489593
E.caballus	57	47.41332774	55.03845009
O.anatinus	22.6	20.05198674	22.07864545
O.mykiss	11	10.82548804	10.96429273
I.punctatus	16	14.80242713	15.754962
C.porcellus	12	5	10.56771474
C.jacchus	22.8	10	20.18096409
P.abelii	55	55	55
A.melanoleuca	36.8	30	35.40863717
N.leucogenys	44.1	44.1	44.1
L.africana	65	53.77643029	62.70352093
C.griseus	2.5	2.5	2.5
O.niloticus	9	9.234712396	9.048025015
S.harrisii	13	12.41626367	12.88056044
O.garnettii	20	20	20
P.paniscus	55	41.5	52.23773556
P.anubis	25.2	25.2	25.2
S.boliviensis	30.3	26.17647295	29.45627614
G.gorilla	60.1	49.87902998	58.00866504
O.orca	90	73.66112578	86.65686732
T.truncatus	51.6	45	50.24955961
T.manatus	69	56.95798157	66.53605635
O.rosmarus	35	29.91479571	33.95950526
C.simum	45	37.8686739	43.54084382
D.novemcinctus	22.3	19.8133704	21.79120529
O.princeps	7	7.643936757	7.131757304
S.araneus	3.2	4.621463042	3.490848652
O.degus	14	6.5	12.46540865
J.jaculus	7.3	7.882553103	7.419197461
C.cristata	2.5	4.064691569	2.820154954
E.telfairi	19	17.18859059	18.62936357
M.putorius	11.1	10.90502682	11.06010611
H.glaber	31	31	31
M.auratus	3.9	2.5	3.613542947
C.picta	61	50.59487901	58.87098551
I.tridecemlineatus	7.9	7.9	7.9
M.ochrogaster	5.3	5.3	5.3
C.lanigera	17.2	10	15.7267923
M.fascicularis	39	38	38.79538782
C.hircus	20.8	20.8	20.8
X.maculatus	5	6.053161118	5.215489593

M.brandtii	41	34.68712262	39.7083084
B.mutus	20	17.98397841	19.58749743
A.sinensis	65	53.77643029	62.70352093
B.bubalis	34.9	34.9	34.9
M.lucifugus	34	29.11940789	33.00137141
P.sinensis	20	17.98397841	19.58749743
T.chinensis	10	10.03010022	10.00615887
C.ferus	40	33.8917348	38.75017454
V.pacos	25.8	25	25.63631026
L.oculatus	18	16.39320277	17.67122972
L.weddellii	25	25	25
M.davidii	41	34.68712262	39.7083084
N.brichardi	9	9.234712396	9.048025015
C.asiatica	4	5.257773298	4.257355737
E.edwardii	6	6.848548937	6.173623448
P.alecto	20.3	18.22259476	19.87493758
P.maniculatus	8.3	8.677940922	8.377331316
C.mydas	75	61.73030849	72.28485949
P.tigris	26.3	22.99492167	25.62374072
P.macrocephalus	77	68	75.15849038
B.acutorostrata	50	41.845613	48.3315131
A.mexicanus	15	14.00703931	14.79682815
P.bivittatus	20	17.98397841	19.58749743
L.vexillifer	24	24	24
E.europaeus	11.7	11.38225951	11.63498643
O.afer	29.8	25.77877904	28.97720921
C.sabaeus	12	12	12
C.syrichta	16	13	15.38616346
A.carolinensis	7.2	7.803014321	7.323384075
E.fuscus	19	17.18859059	18.62936357
S.partitus	4	5.257773298	4.257355737
C.semilaevis	14	13.21165149	13.83869429
P.reticulata	5	6.053161118	5.215489593
E.przewalskii	36	30.71018352	34.91763912
G.variegatus	18	16.39320277	17.67122972
U.maritimus	43.8	38.2	42.65417179
N.galili	20	17.98397841	19.58749743
R.roxellana	29.5	29.5	29.5
H.leucocephalus	48	40.25483736	46.41524538
F.damarensis	15.5	14.40473322	15.27589508
L.crocea	6	6.848548937	6.173623448
B.bison	33.5	28.72171398	32.52230448
E.lucius	30	25.93785661	29.16883598
C.bactrianus	35.4	35	35.31815513
C.dromedarius	28.4	45	31.79656219
P.vampyrus	20.9	18.69982745	20.4498179
M.nemestrina	37.6	31.98280404	36.45065329
C.angolensis	35.3	30.15341205	34.24694542
M.leucophaeus	39	28	36.74926602
C.atys	26.8	26.8	26.8
A.nancymaae	20	17.98397841	19.58749743
P.coquereli	30	28	29.59077564
M.murinus	18.2	14	17.34062884
F.heteroclitus	4	5.257773298	4.257355737
D.ordii	9.9	9.9	9.9
S.salar	13	12.41626367	12.88056044

E.asinus	47	39.45944954	45.45711153
S.vulgaris	22.9	20.29060309	22.36608561
P.latipinna	5	6.053161118	5.215489593
A.jubatus	20.5	17	19.78385737
C.variegatus	15.2	14.16611688	14.98845492
G.japonicus	7	7.643936757	7.131757304
M.marmota	17.4	15.91597008	17.0963494
N.furzeri	1.1	2.951148621	1.478767556
R.aegyptiacus	22.9	20.29060309	22.36608561
M.natalensis	13	12.41626367	12.88056044
C.imitator	45	45	45
P.nattereri	10	10.03010022	10.00615887
R.bieti	23	23	23
L.calcarifer	20	20	20
C.carpio	47	39.45944954	45.45711153
P.pardus	27.3	22	26.21555544
H.armiger	10	10.03010022	10.00615887
P.olivaceus	12.3	11.8594922	12.20986674
C.canadensis	23.4	11	20.86280896
O.kisutch	5	6.053161118	5.215489593
M.albus	10	10.03010022	10.00615887
L.bergylta	29	29	29
P.vitticeps	12	11.62087585	11.92242658
O.virginianus	23	20.37014187	22.46189899
P.cinereus	22.1	19.65429283	21.59957852
L.striata	10	10.03010022	10.00615887
M.unguiculatus	6.3	6.3	6.3
N.schauinslandi	30	25	28.9769391
E.lutris	27	23.55169315	26.29443442
D.leucas	40	40	40
S.dumerili	15	14.00703931	14.79682815
P.tephrosceles	18.4	12.1	17.11094326
A.ocellaris	7	7	7
S.lalandi	12	11.62087585	11.92242658
T.triunguis	50.6	42.32284569	48.90639341
O.melastigma	0.25	2.275068975	0.6643537784
O.tshawytscha	9	9.234712396	9.048025015
T.gelada	36	20.8	32.88989486
C.ursinus	25	25	25
V.vulpes	21.3	12	19.39710672
A.calliptera	10	10.03010022	10.00615887
C.auratus	41	34.68712262	39.7083084
M.armatus	18	16.39320277	17.67122972
A.testudineus	8	7	7.79538782
U.parryii	10	9	9.79538782
U.arctos	40	33.8917348	38.75017454
P.textilis	20	17.98397841	19.58749743
E.electricus	12.6	12.09810855	12.4973069
S.obliquidens	46	38.66406172	44.49897767
Z.californianus	35.7	30	34.53371057
V.ursinus	30	25.93785661	29.16883598
M.flaviventris	21.2	18.93844379	20.73725805
X.couchianus	4	5.257773298	4.257355737
E.jubatus	32.8	28.1649425	31.85161078
P.ranga	5.5	5.5	5.5
P.discolor	9	9.234712396	9.048025015

P.flavescens	12	12	12
P.muralis	10	10.03010022	10.00615887
E.calabaricus	20	17.98397841	19.58749743
P.leucopus	7.9	8.359785794	7.994077774
D.clupeoides	14	13.21165149	13.83869429
M.monoceros	50	50	50
C.gobio	5	6.053161118	5.215489593
O.nerka	8	8.439324576	8.089891159
S.trutta	38	32.30095916	36.83390683
S.suricatta	20.6	12.5	18.94264134
S.fasciatus	3	3	3
S.orbicularis	4	4	4
Ly.canadensis	26.8	23.39261558	26.10280765
Lo.canadensis	27	23.55169315	26.29443442
G.morhua	25	21.96091751	24.37816671
S.aurata	11	11	11
S.habroptila	60	49.79949119	57.91285165
C.chanos	15	14.00703931	14.79682815
G.melas	60	60	60
C.parvulus	15	14.00703931	14.79682815
S.lucioperca	16	16	16
M.coucha	3	3	3
O.aureus	3	4.462385478	3.299221881
H.moloch	45	37.8686739	43.54084382
S.apella	46	40	44.77232692
M.erminea	12.5	12.01856976	12.40149351
P.vitulina	47.6	39.93668223	46.03199184
E.spectabile	4	4	4
X.hellerii	5	6.053161118	5.215489593
P.sinus	12	11.62087585	11.92242658
C.abingdonii	150	121.384395	144.1448987
R.rattus	4.2	5.416850862	4.448982508
C.ustulatus	11	10.82548804	10.96429273
R.ferrumequinum	30.5	26.33555052	29.64790291
T.francoisi	26.3	22.99492167	25.62374072
G.seraphini	20	17.98397841	19.58749743
T.bernacchii	10	10.03010022	10.00615887
P.guttatus	15.6	14.48427201	15.37170846
A.niloticus	6.7	7.405320411	6.844317147
C.lumpus	13	12.41626367	12.88056044
H.hippoglossus	90	73.66112578	86.65686732
N.celidotus	7	7.643936757	7.131757304
E.cragini	3	4.462385478	3.299221881
M.leonina	20	17.98397841	19.58749743
Z.vivipara	6	6.848548937	6.173623448
H.stenolepis	42	35.48251044	40.66644225
A.anguilla	88	72.07035014	84.74059961
S.maximus	26	22.75630533	25.33630056
M.saxatilis	30	25.93785661	29.16883598
O.keta	7	7.643936757	7.131757304
H.grypus	42.9	36.19835948	41.52876272
O.torridus	4.6	5.73500599	4.83223605
M.molossus	5.6	6.530393809	5.790369906
M.myotis	37.1	31.58511013	35.97158636
P.kuhlii	8	8.439324576	8.089891159
M.cyprinoides	44	37.07328608	42.58270996

T.vulpecula	15.9	14.72288835	15.65914862
B.musculus	110	85	104.8846955
A.jamaicensis	19.2	17.34766816	18.82099034
P.pungitius	5	6.053161118	5.215489593
T.occidentalis	7	7.643936757	7.131757304
C.didactylus	36.8	31.34649378	35.6841462
A.platyrhynchos	29.1	25.22200757	28.30651551
C.tularosa	5	6.053161118	5.215489593
A.amphibius	2.5	4.064691569	2.820154954
D.coriacea	30	25.93785661	29.16883598
M.salmoides	23	20.37014187	22.46189899
T.aculeatus	49.5	41.44791909	47.85244617
S.namaycush	50	41.845613	48.3315131
H.hyaena	25	21.96091751	24.37816671
C.tigris	15.3	14.24565566	15.08426831
M.reevesii	24.2	21.32460725	23.61165962
P.promelas	2	3.666997659	2.341088026
P.senegalus	34	29.11940789	33.00137141
P.fluviatilis	22	22	22
S.diagramma	7	7.643936757	7.131757304
X.gladius	12	11.62087585	11.92242658
G.aculeatus	8	8.439324576	8.089891159
O.dammah	27.5	23.94938706	26.77350134
R.temporaria	27	23.55169315	26.29443442
P.yagouaroundi	18.6	16.87043546	18.24611003
O.curzoniae	1.58	3.332934775	1.938671806
C.carcharias	50	41.845613	48.3315131
M.oregoni	1.25	3.070456794	1.622487634
T.guttata	12	11.62087585	11.92242658
V.lagopus	16.3	15.04104348	16.04240216
C.undulatus	21	18.77936623	20.54563128
C.clupeaformis	50	41.845613	48.3315131
A.sapidissima	13	12.41626367	12.88056044
T.maccoyii	20	20	20
P.leopardus	26	22.75630533	25.33630056
D.spectabilis	12.7	12.17764733	12.59312028
P.leo	28	28	28
P.tetrazona	10	10.03010022	10.00615887
P.bengalensis	17	15.59781495	16.71309586
C.plagiosum	25	21.96091751	24.37816671
C.elaphus	31.5	27.13093834	30.60603677
G.affinis	3	4.462385478	3.299221881
S.chuatsi	9	9.234712396	9.048025015
N.vison	11.4	11.4	11.4
T.albacares	9	9	9
V.komodoensis	62	51.39026683	59.82911936
G.agilis	6.1	6.928087719	6.269436834
M.mutica	22.1	19.65429283	21.59957852
L.geoffroyi	23	20.37014187	22.46189899
L.catta	37.3	31.74418769	36.16321313
U.americanus	34	32	33.59077564
P.hastatus	18	16.39320277	17.67122972
M.angustirostris	18	16.39320277	17.67122972
M.meles	18.6	16.87043546	18.24611003
M.dolomieu	26	22.75630533	25.33630056
O.gorbuscha	3	4.462385478	3.299221881

S.argus	12	11.62087585	11.92242658
M.monax	14	14	14
E.quagga	38	32.30095916	36.83390683
H.transpacificus	1	2.871609839	1.38295417
L.rufus	32.3	32.3	32.3
S.carolinensis	23.6	20.84737456	23.03677931
L.lutra	18.2	16.55228034	17.86285649
P.africanus	30	25.93785661	29.16883598
P.viverrinus	17.2	15.75689252	16.90472263
A.alosa	10	10.03010022	10.00615887
P.longimembris	8.3	8.677940922	8.377331316
M.glareolus	4.9	5.973622336	5.119676207
C.caretta	77	63.32108413	74.2011272
E.fuscoguttatus	42	35.48251044	40.66644225
A.gentilis	22	19.57475405	21.50376514
E.maximus	79.6	65.38909246	76.69227522
M.thibetana	27	23.55169315	26.29443442
L.rohita	10	10.03010022	10.00615887
P.roborovskii	4.8	5.894083554	5.023862821
D.labrax	15	15	15
C.idella	21	18.77936623	20.54563128
A.flavipes	3.9	5.178234516	4.161542351
A.sylvaticus	6.3	7.087165283	6.461063605
C.gibelio	10	10.03010022	10.00615887
B.taxicolor	21.9	19.49521527	21.40795175
S.japonicus	18	16.39320277	17.67122972
P.platessa	50	41.845613	48.3315131
S.bombifrons	13	12.41626367	12.88056044
C.gariepinus	15	14.00703931	14.79682815
N.coucang	25.8	22.59722776	25.14467379
I.furcatus	21	18.77936623	20.54563128
B.bombina	20	17.98397841	19.58749743
C.canorus	12.9	12.33672489	12.78474705
P.pygmaeus	59	49.00410337	56.9547178
A.fimbria	114	92.75043345	109.6520799
A.phoeniceus	20	17.98397841	19.58749743
G.americana	40	33.8917348	38.75017454
E.macularius	28.5	24.74477488	27.7316352
M.anguillicaudatus	10	10.03010022	10.00615887
S.syndactylus	43	36.27789826	41.62457611
N.procyonoides	16.6	15.6	16.39538782
P.obesus	3.2	4.621463042	3.490848652
S.fontinalis	24	21.16552969	23.42003285
G.chalcogrammus	15	14.00703931	14.79682815
H.amphibius	61.2	50.75395658	59.06261228
C.nivalis	3	4.462385478	3.299221881
C.intestinalis	2	3.666997659	2.341088026
A.prasina	15	14.00703931	14.79682815
D.bicornis	57	47.41332774	55.03845009
S.solea	27	23.55169315	26.29443442
N.nebulosa	19.8	17.82490085	19.39587066
P.atricapillus	12.4	11.93903098	12.30568012
K.breviceps	17	17	17
M.lutreola	8.4	8.757479704	8.473144702
P.eremicus	7.4	7.962091885	7.515010846
C.striata	20	10	17.9538782

M.nigripes	9.4	9.552867524	9.431278557
C.carassius	43	36.27789826	41.62457611
M.daubentonii	28	24.34708097	27.25256827
H.mexicanus	11.6	11.30272073	11.53917304
D.delphis	25	25	25
G.macrocephalus	18	16.39320277	17.67122972
M.densirostris	27	27	27
H.binoei	13.6	12.89349637	13.45544075
P.onca	28	24.34708097	27.25256827
A.sagrei	8	8.439324576	8.089891159
L.mixtus	20	17.98397841	19.58749743
D.dama	27	23.55169315	26.29443442
E.glacialis	67	67	67
B.javanicus	27.1	23.63123193	26.3902478
C.saira	2	3.666997659	2.341088026
C.striatus	11.5	11.22318194	11.44335965
L.europaeus	10.7	10.58687169	10.67685257
P.flesus	15	15	15
S.scombrus	17	17	17
O.eperlanus	10	10.03010022	10.00615887
S.pilchardus	15	14.00703931	14.79682815
E.multicarinata	9.8	9.871022652	9.8145321
E.encrasicolus	3	4.462385478	3.299221881
C.aspera	9.9	9.950561434	9.910345485
A.rostrata	50	41.845613	48.3315131
D.novaehollandiae	16.6	15.27965982	16.32984232
P.phocoena	20.4	13	18.88586986
M.reevesi	23.2	20.52921943	22.65352576
S.bilineata	6	6.848548937	6.173623448
T.scincoides	26.6	23.23353802	25.91118087
A.calva	30	25.93785661	29.16883598
A.cygnoides	20	17.98397841	19.58749743
O.mordax	7	7.643936757	7.131757304
E.macquarii	20.9	18.69982745	20.4498179
T.thynnus	15	14.00703931	14.79682815
P.crassidens	62.5	51.78796074	60.30818629
A.striatus	19.9	17.90443963	19.49168404
E.robustus	77	63.32108413	74.2011272
A.acuta	27.4	23.86984828	26.67768796
S.camelus	50	41.845613	48.3315131
C.sumatraensis	18.5	16.79089668	18.15029664
P.breviceps	17.8	16.23412521	17.47960294
E.jacksoni	10	10.03010022	10.00615887
P.waltl	19.5	17.5862845	19.1084305
E.rufifrons	14.5	13.6093454	14.31776122
O.canadensis	20.6	18.4612111	20.16237774
A.mexicanum	17	15.59781495	16.71309586
H.albicilla	42	35.48251044	40.66644225
D.merriami	9.7	9.79148387	9.718718714
M.yumanensis	14	13.21165149	13.83869429
