# Supplementary material for: Structural and mechanistic diversity in p53-mediated regulation of organismal longevity across taxonomical orders
Source: PLoS Comput Biol. 2025 May 2;21(5):e1012382. doi: 10.1371/journal.pcbi.1012382 (PMC12068700; doi:10.1371/journal.pcbi.1012382)
Supplement: S3 File — (PDF) [file pcbi.1012382.s004.pdf]

			log(LR)						
	Organism	Average lifespan	p53_Klf4	Pras40_Akt	p53_Npm1	p53_mdm2	p53_Smad2	p53_Smad3	Rpl11_mdm2
Primates	Microcebus murinus	14	-0.314	0.313	0.306	0.443	0.339	0.345	1.704
	Sapajus apella	40	0.511	0.37	0.537	0.518	0.491	0.456	1.699
	Gorilla gorilla gorilla	47	0.751	0.32	0.388	0.833	0.645	0.434	1.692
	Pan troglodytes	56	0.787	0.362	0.508	0.913	1.12	1.094	1.63
	Homo sapiens	76	0.853	0.473	0.382	0.877	1.001	0.268	1.692
	Macaca mulatta	26	-0.177	0.37	0.333	0.603	0.619	0.34	1.679
	Saimiri boliviensis boliviensis	17.5	0.37	0.376	1.303	0.915	0.738	0.379	1.72
	Theropithecus gelada	20.8	-0.416	0.354	-0.433	0.585	-0.149	0.087	1.637
	Rhinopithecus bieti	23	-0.028	0.581	0.435	0.656	0.886	0.439	1.72
	Papio anubis	25.2	0.002	0.354	0.342	0.514	0.838	0.446	1.633
Perciformes	Perca flavescens	12	-1.028	-0.719	-0.08	-0.232	-0.196	-0.089	1.177
	Dicentrarchus labrax	15	-1.231	-1.047	-0.827	-0.282	0.022	0.512	2.006
	Sander lucioperca	16	-1.157	-0.799	-1.075	-0.259	-0.183	-0.177	2.001
	Perca fluviatilis	22	-1.208	-1.094	-0.984	-0.461	-0.196	-0.22	1.309
	Labrus bergylta	29	-1.167	-0.993	-0.185	-0.356	0.239	0.707	2.082
	Anabas testudineus	7	-1.339	-1.046	-0.035	-0.975	-0.124	-0.121	1.129
	Amphiprion percula	18	-1.229	-1.001	-0.09	-0.316	-0.157	-0.098	2.009
	Sparus aurata	11	-1.122	-0.98	-0.274	-0.347	0.014	0.075	1.56
